# Composition‐Dependent Wide‐Range Tunability of Optical and Electronic Properties in SnS_x_Se_(2‐x)_ Alloy Nanosheets

**DOI:** 10.1002/smll.202512066

**Published:** 2025-12-31

**Authors:** Nicolas J. Diercks, Rebekah A. Wells, Shixin Liu, Tian Carey, Jack Doran, Joseph Neilson, YeonJu Kim, Jun‐Ho Yum, Goutam Ghosh, Hannah Johnson, Laurens D. A. Siebbeles, Jonathan N. Coleman, Kevin Sivula

**Affiliations:** ^1^ Laboratory For Molecular Engineering of Optoelectronic Nanomaterials Institute of Chemical Sciences and Engineering École Polytechnique Fédérale de Lausanne (EPFL) Lausanne Switzerland; ^2^ Laboratory For Chemical Physics of Low‐Dimensional Nanostructures, School of Physics Trinity College Dublin Dublin Ireland; ^3^ Chemical Engineering Department Delft University of Technology Delft, HZ The Netherlands; ^4^ Advanced Materials Research Toyota Motor Europe Zaventem Belgium

**Keywords:** 2D, alloy, optoelectronic, printable, solution‐Processing

## Abstract

Isovalent alloying in printable metal dichalcogenide nanomaterials enables precise, application‐targeted property tuning. However, a scalable platform offering broad optical and electrical tunability has so far remained elusive. Herein, we establish a powder‐based, solution‐processed route to access the full domain of SnS_x_Se_(2‐x)_ alloy nanosheets, providing control over a wide range of properties through chalcogenide composition. The n‐type nanosheet alloy series shows a wide spread in optical and in‐plane electrical properties, ranging from 1.67 eV and low bandgap metallic‐like behavior for 2D SnSe_2_, to 2.46 eV and wide bandgap semiconducting behavior with high‐resistivity for 2D SnS_2_. The out‐of‐plane conductivity is also tunable, showing nonmonotonic behavior with an optimal chalcogenide ratio of x = 1.2 – 1.6. Using photoelectrochemistry as an example, we highlight how the interplay of these tunable properties enables optimized performance for targeted applications. The exceptional range of tailorable properties reported here provides a roadmap for tuning these alloys, thereby opening avenues for their potential application in a multitude of fields.

## Graphical abstract

A scalable, solution‐processed route unlocks the full SnS_x_Se_(2–x)_ alloy spectrum, delivering finely tunable optical and electronic properties—from metallic‐like SnSe_2_ to wide‐bandgap SnS_2_. Composition‐guided control over in‐ and out‐of‐plane transport enables performance‐optimized photoelectrochemistry, charting a versatile roadmap for designer printable dichalcogenides.

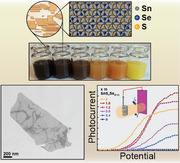

## 1 Introduction

Layered semiconductors with chemical formula MX_2_ (M = metal, X = chalcogen) are a distinct class of materials whose optical, electrical, mechanical, and thermal responses can be tuned by their thickness from bulk crystals down to the monolayer limit. Their weak interlayer van der Waals coupling facilitates the isolation of atomically thin crystals with exceptional properties. As the field pivots toward scalable, solution‐processable, and printable routes compatible with large‐scale manufacturing, identifying 2D platforms that enable a greater range of tunable properties is essential.

The alloying of materials is an established route toward combining and optimizing material functionalities. This method has been well documented in transition metal dichalcogenides (TMDs) such as MoS_2_, WS_2_, MoSe_2_, and WSe_2_ in order to achieve alloys such as MoWS_2_, MoWSe_2_, MoSSe, WSSe, and MoWSSe with composition‐tunable properties [1, 2, 3, 4, 5, 6, 7, 8, 9]. These studies have mainly leveraged bottom‐up techniques like chemical vapor deposition (CVD) [1, 2, 3, 4, 5], chemical vapor transport (CVT) [6, 7], atomic layer deposition (ALD) [8], which often experience challenges in controlling the exact composition, building up layers, and are not suitable for large‐quantity production. Importantly, it has been recently shown that alloyed TMDs can alternatively be made using powder‐based, solution‐processable approaches, making them suitable for 2D printing and desirable for large‐scale electronic and optoelectronic devices [10]. The remarkable progress achieved with TMDs has already enabled fine‐tuning (*e.g*., down to tenths of an electron Volt) of properties within a limited range (*e.g*., bandgaps between 1.5 and 2 eV) [4, 10], which underscores the promise of 2D semiconductors and motivates the exploration of new material families that can afford an even broader spectrum of tunability.

Among emerging candidates, the post‐transition metal dichalcogenides (p‐TMDs), notably tin disulfide (SnS_2_) and tin diselenide (SnSe_2_), have independently attracted intense interest in a diverse portfolio of applications, including photoelectrochemical water splitting, photodetectors, memristive switching devices, and thermoelectric devices [11, 12, 13, 14, 15, 16, 17, 18, 19]. Similar to TMDs, p‐TMDs are layered materials with chemical formula MX_2_; however, they possess the CdI_2_‐type hexagonal structure wherein Sn is octahedrally coordinated by six chalcogen atoms (S or Se) [20]. Crucially, solution‐phase routes such as exfoliation via electrochemical intercalation have been proven effective for producing dispersions of high‐quality SnS_2_ and SnSe_2_ nanosheets [21, 22]. Moreover, these materials are made up of inexpensive, earth‐abundant elements, making them suitable for large‐scale production and processing [11, 23, 24].

Electrically, SnS_2_ is typically reported as a wide‐bandgap semiconductor (bulk ∼2.07 eV) [25], while SnSe_2_ is substantially more conductive with a smaller bandgap (∼1 eV) [19]. In combination with their crystal compatibility, their optoelectronic differences open an attractive route for isovalent alloying to enable continuous tuning of optical absorption, electronic structure, and transport over a broad range. Such tunability is practical for bandgap engineering, optimizing charge transfer at interfaces, and precise spectral matching. Indeed, previous works in which SnS_x_Se_(2‐x)_ nanosheets were mechanically exfoliated from bulk crystals or grown via CVT demonstrated high‐performance photodetectors and composition‐tunable field‐effect transistors, highlighting the promise of these materials in optoelectronic devices [26, 27, 28]. However, such techniques allow only limited control over the final chalcogenide composition, and therefore, a study spanning the full compositional range has so far not been performed. Additionally, these methods are not suitable for large‐area devices or scaled‐up production, particularly in the case of printable electronics. Thus, there exists a need to develop an alternative solution‐processable technique capable of accessing the full range of compositions and material properties while being amenable to post‐production processes such as printing.

In this work, we present a framework for the solution‐processable production of dispersions of alloyed 2D SnS_x_Se_(2‐x)_ nanosheets from commercially available starting materials. We outline scalable synthesis and dispersion protocols, establish compositional control, and map composition‐dependent optical, electrical, and optoelectronic properties across the full S‐Se range. Finally, we demonstrate the printability of these nanosheets by employing a scalable liquid‐liquid interfacial self‐assembly technique to deposit thin films of SnS_x_Se_(2‐x)_ nanosheets on a variety of substrates [29, 30]. Collectively, our results position alloyed SnS_x_Se_(2‐x)_ nanosheets as a versatile and scalable platform for a new generation of highly tunable 2D materials.

## 2 Results and Discussion

**FIGURE 1:**
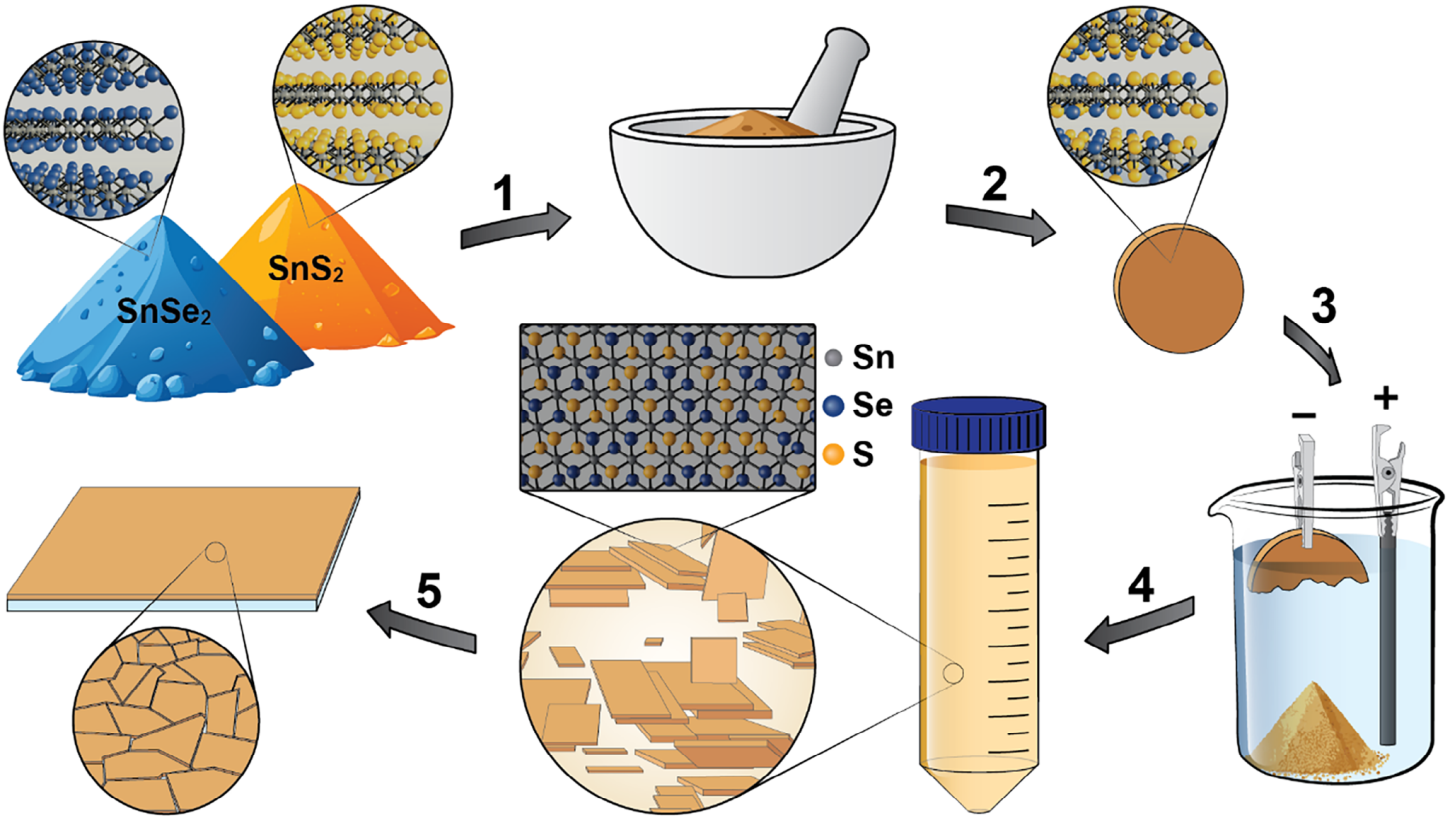
Schematic procedure for preparing SnS_x_Se_(2‐x)_ alloy nanosheet films. (1) Bulk powders of SnS_2_ and SnSe_2_ are mixed and mechanically ground together. (2) The ground powder is pressed into a pellet and sintered in a closed system. (3) The sintered pellet acts as the cathode in tetraheptylammonium bromide (THA^+^ Br^−^) electrolyte with a glassy carbon anode. THA^+^ intercalation (driven by an applied bias) leads to the eventual detachment of intercalated material. (4) The intercalated powder is collected, rinsed, gently agitated, and centrifuged at low speed to remove any bulk material. The result is a solution of exfoliated, alloyed SnS_x_Se_(2‐x)_ nanosheets that (5) can be used to make nanosheet‐based thin film devices.

Our scalable route for preparing SnS_x_Se_(2‐x)_ nanosheet thin films is schematically presented in Figure 1 and described in detail in the Experimental Section. Bulk powders of SnSe_2_ and SnS_2_ are mixed in the desired ratio and mechanically ground together. The resulting powder mixture is pressed and sintered to form a mechanically robust and electrically conductive polycrystalline pellet. Figure S1 shows photographs of the different SnS_x_Se_(2‐x)_ pellets with increasing sulfur content (*x* = 0 to 2). As visible from scanning electron microscopy (SEM) images in Figure S2, the sintering leads to the growth of large crystallites of alloyed material. The sintering conditions are tailored to the specific chalcogen species, where higher sintering temperatures are required for sulfur (S) compared to selenium (Se) [10, 31]. For example, pure SnSe_2_ (*x* = 0) is heated at 600°C for 48 h, whereas pure SnS_2_ (*x* = 2) is heated at 650°C for 12 h. By maintaining a closed sintering system, the initial stoichiometric ratio is preserved, enabling the formation of a single material alloy of the desired mixed S/Se ratio. The incorporation of both Se and S into one alloyed phase in the bulk, sintered pellets, is confirmed by both powder X‐ray diffraction (PXRD, see Figure S3; Note S1) and Raman spectroscopy (see Figure S4; Note S2).

Electrochemical intercalation of tetraheptylammonium (THA^+^) into the interlayer spaces in the bulk pellet, followed by exfoliation using low‐power bath sonication and low‐speed centrifugation to remove unexfoliated bulk material, yields a dispersion of SnS_x_Se_(2‐x)_ nanosheets. It should be noted that these dispersions contain a range of nanosheet sizes and nanosheet thicknesses. Different flake sizes may be selected according to the needs of the intended application [24, 32, 33]. Using film formation techniques, such as liquid‐liquid interfacial self‐assembly (LLISA) [29, 34], high‐quality thin films of aligned nanosheets can be deposited onto various substrates.

To confirm successful exfoliation and alloying at the atomic level, we examined a prototypical SnSSe (1:1, S:Se, molar feed ratio for pellet formation) nanosheet using transmission electron microscopy (TEM) and scanning transmission electron microscopy (STEM), as shown in Figure S5. As visible from the TEM image in Figure S5a, crystalline nanosheets are produced using the electrochemical pellet intercalation (ECPI)‐exfoliation method. In addition, STEM energy dispersive X‐ray (EDX) in Figure S5b–e confirms the presence and homogeneous distribution of the different elements (Sn, Se, and S) over the entire nanosheet area, supporting the absence of chalcogenide‐specific domains.

**FIGURE 2:**
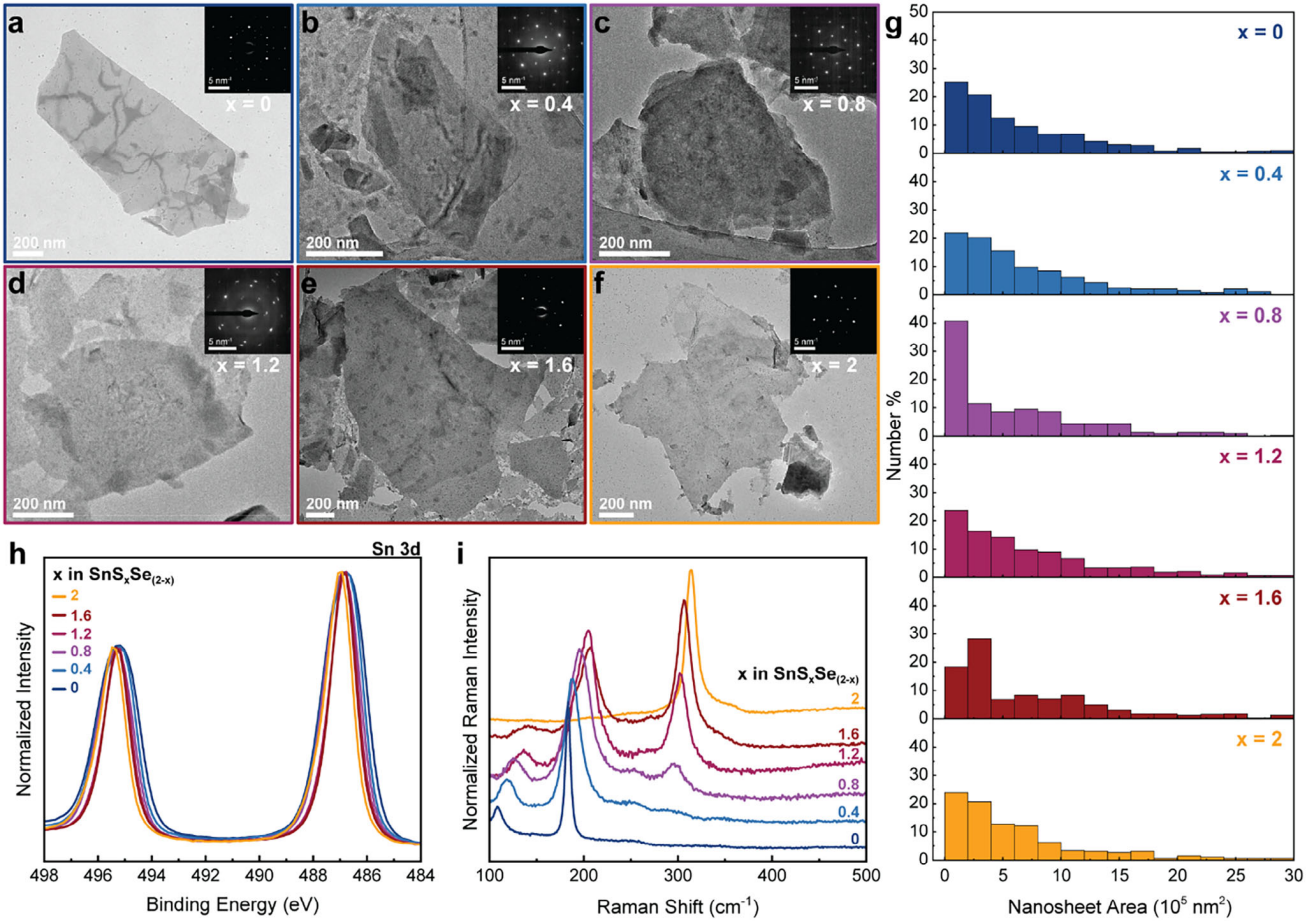
(a–f) Bright‐field TEM images of SnS_x_Se_(2‐x)_ alloys nanosheets of different S content (*x* = 0 to 2). (g) Sheet area size distribution of nanosheets of the different compositions in (a–f) in percentage of the overall nanosheet number. (h) Normalized XPS spectra of the Sn 3d peaks of SnS_x_Se_(2‐x)_ nanosheet thin films with varying sulfur composition (*x* = 0 to 2). (i) Normalized Raman spectra of SnS_x_Se_(2‐x)_ nanosheet thin films with varying sulfur composition (*x* = 0 to 2).

Having demonstrated the possibility of alloyed SnS_x_Se_(2‐x)_ nanosheet formation using our approach with *x* = 1, we expanded it to encompass the full range of S content (*x* = 0 to 2). Figure 2a–f shows TEM images for six different nanosheet compositions with S content ranging from *x* = 0 to 2. The TEM images show that nanosheets can be acquired for the entire range of compositions. Importantly, the selected area electron diffraction (SAED) patterns for each composition (insets Figure 2a–f) confirm the crystallinity of the nanosheets across the range of selected S to Se ratios. Figure 2g shows the nanosheet area distribution for the selected compositions and suggests a dependency of nanosheet area on composition, wherein the likelihood of finding a large nanosheet (>5 10^5^ nm^2^) appears to be greater for higher sulfur content. The average nanosheet area in relation to nanosheet composition (presented in Figure S6a) further highlights this, where the average nanosheet size appears to increase with sulfur content. We hypothesize that this difference in nanosheet area might stem from the different intercalation speeds during the ECPI process, where an increase in sulfur content leads to slower intercalation speeds, most likely due to reduced conductivity of the sintered SnS_x_Se_(2‐x)_ pellet. This reduced intercalation speed potentially leads to milder intercalation, resulting in fewer material fractures and, consequently, larger nanosheets after exfoliation. A similar composition‐dependence of the nanosheet thickness could not be confirmed, as visible from the average nanosheet thickness for each composition presented in Figure S6b.

To confirm the composition of the nanosheets, we examine the X‐ray photoelectron spectroscopy (XPS) Sn 3d peak position for thin films of nanosheets (Figure 2h). The signal shows the expected split of 8.4 eV, and peak positions around 495 eV and 487 eV for the Sn 3d^5/2^ and 3d^1/2^ signal, respectively. With increasing S content, the Sn 3d signal gradually shifts to higher binding energies, in line with the small increase in electronegativity of S compared to Se. Similarly, the S 2p and Se 3d XPS signals, depicted in Figure S7a,b, show a distinct evolution in shape and position with changing chalcogenide composition. The Raman spectra in Figure 2i demonstrate the same trend in Raman signal in nanosheet films as for the bulk sintered pellets (Figure S4). The *x* = 0 nanosheet film only exhibits the SnSe_2_‐specific E_g_ and A_1g_ modes at 109.3 and 184.4 cm^−1^, respectively, while the *x* = 2 film only shows the SnS_2_ A_1g_ at 313.8 cm^−1^. For samples with mixed chalcogenide composition, the Sn‐Se E_g_ and A_1g_ modes shift toward higher wavenumbers with increasing S content. Meanwhile, the signal intensity ratio between Sn‐Se E_g_ and A_1g_ modes decreases for reduced Se content. Starting from the *x* = 0.8 sample, the Sn‐S A_1g_ mode starts emerging, which increases in intensity and shifts to higher wavenumbers with increasing S content. This similarity in trends for peak intensity ratios and shifts over the range in both pellets and thin films further supports the successful translation of the chalcogenide feed ratio from bulk to nanosheet.

**FIGURE 3:**
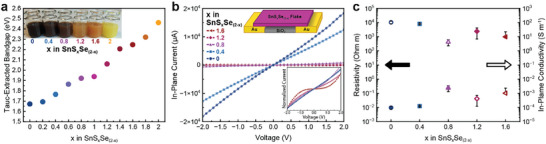
(a) Tauc‐extracted bandgaps of SnS_x_Se_(2‐x)_ nanosheets with changing S content (*x * =  0 to 2). The inset shows a photograph of 6 different SnS_x_Se_(2‐x)_ nanosheet dispersions in NMP. (b) In‐plane current measurements for single SnS_x_Se_(2‐x)_ nanosheets of different composition (*x * =  0 to 1.6). The top inset image shows a schematic of the device architecture used for these measurements. The bottom inset shows the normalized current for each composition. (c) Mean resistivity (closed icons) and in‐plane conductivity (open icons) values based on four devices calculated for the different SnS_x_Se_(2‐x)_ compositions. Error bars show standard deviation from mean.

Having established a route to make the full range of chalcogenide ratios in SnS_x_Se_(2‐x)_ nanosheets, we further examined the optical and electrical nanosheet properties in dispersions as well as thin films with an eye toward devices. From the normalized UV‐vis spectra of nanosheet dispersions depicted in Figure S8, a shift in the peak absorption of the nanosheets to higher wavelengths is observed for reducing S content. Using a Tauc analysis (see Figure S9), optical bandgaps can be extracted and estimated for each composition as shown in Figure 3a. Notably, each composition possesses an indirect bandgap, consistent with pure SnS_2_ and SnSe_2_. Furthermore, the expected thickness‐tunable bandgap of the materials is evidenced by the larger bandgap values observed for thin SnS_2_ and SnSe_2_ compared to values reported for bulk single crystals [20, 25, 35]. The bandgap increases continuously with increasing S content, as expected from the UV‐vis characteristics. With increasing S to Se ratio, the size of the bandgap increases from 1.67 eV in SnSe_2_ (*x* = 0) to 2.46 eV for SnS_2_ (*x* = 2). This impressive range of optical properties is further highlighted by the visual appearance of the nanosheet dispersions, as shown in the inset in Figure 3a.

Given the precise control that can be exerted over the optical properties of these materials as a function of atomic composition, a similar level of control is expected for the electrical properties. In particular, as the bandgap of the materials increases with S content, the charge carrier density, and therefore conductivity, of the material is expected to decrease. To explore this, thin films of SnS_x_Se_(2‐x)_ nanosheets were deposited onto pre‐patterned Fraunhofer (gen 4) electronic testing chips using a previously described liquid‐liquid interfacial self‐assembly [29] (LLISA) printing technique (three depositions). Typical current‐voltage (*I*‐*V*) curves for the 2.5 µm channel length devices are shown in Figure 3b. The *I*–*V* curves show decreasing current for increasing S content, consistent with decreasing material conductivity as a result of decreased charge carrier concentration. Indeed, a reliable *I*‐*V* curve for SnS_2_ could not be measured as the current was below the detection limit of the apparatus, suggesting highly insulating behavior consistent with the wide bandgap calculated in Figure 3a.

The bottom inset in Figure 3b shows the normalized current for each composition. Interestingly, curvature can be observed from *x* = 0.8 onwards, with strong curvature appearing for S‐rich materials (*x* > 1) and increasing with S content. In contrast, Se‐rich (*x* < 1) materials give linear *I*–*V* curves, consistent with more metallic materials. We note that the curvature could also be in combination with increased contact resistance due to band alignment and/or Schottky barriers at the material‐gold interface. This may open avenues to tune such interfaces based on electrode material and alloy composition.

Based on the geometry of the devices, the in‐plane resistivity and conductivity of the materials can be calculated, as shown in Figure 3c. Notably, only 2.5 µm channel length devices (four per composition) were examined in order to minimize electrical scatter and increase the likelihood of a bridging event wherein a single nanosheet is contacted by both electrodes (see optical microscope images of the corresponding device channels provided in Figure S10). This was done to minimize the effects of nanosheet junctions typically present in printed nanosheet devices. Following the trends observed in the *I*–*V* curves, the Se‐rich materials display low resistivities and higher conductivities, while the S‐rich materials yield high resistivities and low conductivities, consistent with conductive and more insulating materials, respectively. Once again, an inflection point in material behavior can be observed for S content greater than *x* = 1.

To determine the estimated charge carrier concentration of these materials, the in‐plane field effect mobility of the materials was measured for S contents of *x* ≤ 1.2 and lower. Typical n‐type transfer curves are shown in Figure S11. For materials with S content *x* ≥ 1.2, a reliable in‐plane mobility could not be extracted. As expected, Se‐rich materials display reasonable mobilities before dropping off sharply for S content with *x* ≥ 1 (Figure S12a). Accordingly, the estimated charge carrier concentration follows the same trend (Figure S12b). In contrast, materials with S content *x* ≥ 0.8 display a higher on/off ratio compared to the Se‐rich materials (Figure S12a).

Next, the optoelectronic properties of the nanosheets are probed via photoelectrochemistry, an ideal medium to explore the result of simultaneously tuning optical (*e.g*., optical bandgap) and electrical (*e.g*., conductivity) properties. The large range of bandgaps and, by extension, band alignments, necessitates an electrochemical reaction whose energy lies well within the bandgap of all materials. For this reason, oxidation of iodide to triiodide (I^−^ → I_3_^−^) was chosen as a suitable and simple reaction with a well‐studied mechanism [36].

**FIGURE 4:**
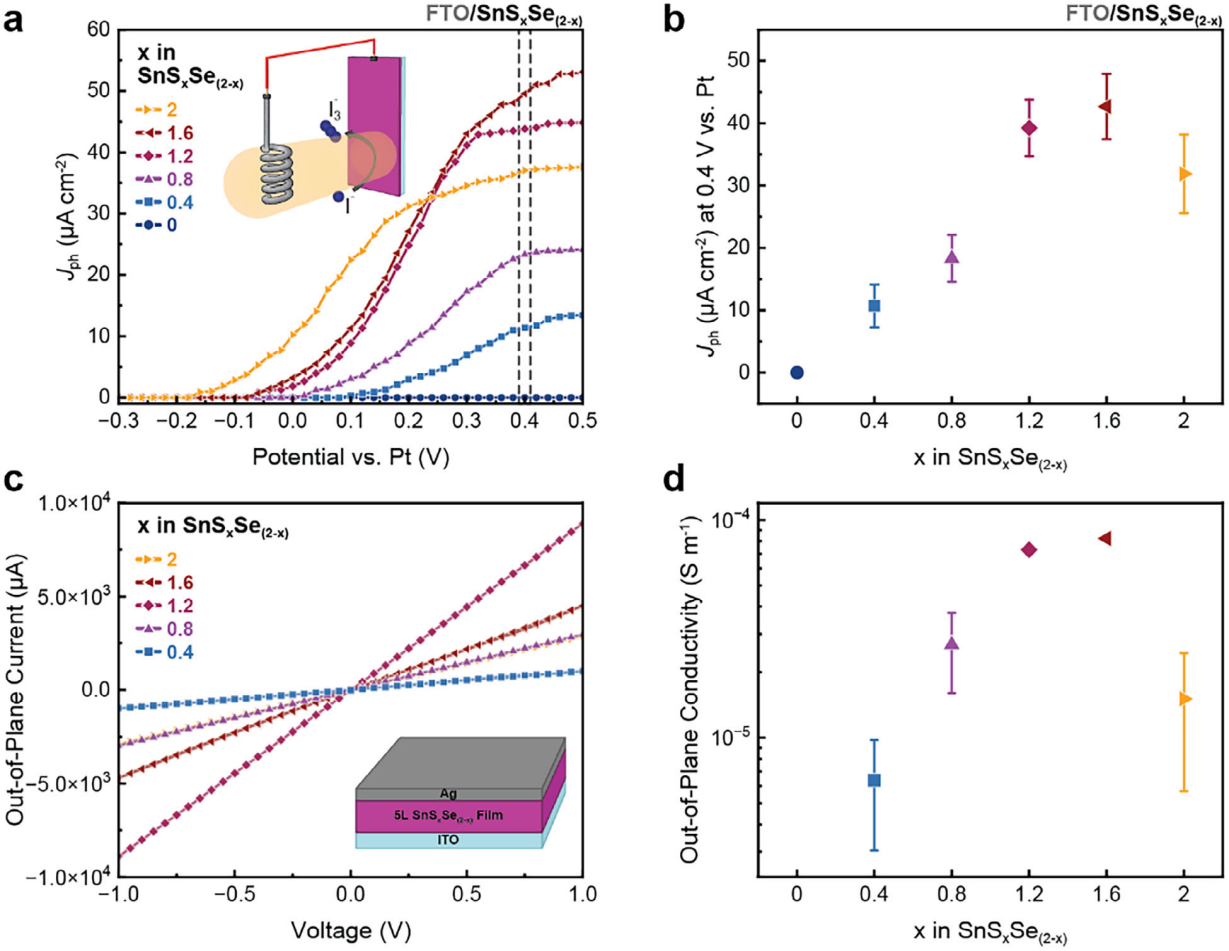
(a) Photocurrent density extracted from LSV measurements of FTO/SnS_x_Se_(2‐x)_ nanosheet devices for nanosheets of different compositions (*x* = 0 to 2) for iodide oxidation in acetonitrile under intermittent 1‐sun illumination. (b) Extracted photocurrent densities (average + standard deviation) of FTO/SnS_x_Se_(2‐x)_ nanosheet devices for iodide oxidation at 0.4 V vs. Pt. (c) Out‐of‐plane current measurements for ITO/5‐deposition layer SnS_x_Se_(2‐x)_ nanosheet film/Ag devices for different SnS_x_Se_(2‐x)_ nanosheet compositions (*x* = 0.4 to 2). The inset shows the device architecture used for these measurements. (d) Extracted composition‐dependent out‐of‐plane conductivities for SnS_x_Se_(2‐x)_ nanosheet networks. Error bars show the standard deviation from the mean.

Photoelectrochemical (PEC) devices were made by coating fluorine‐doped tin oxide (FTO) with SnS_x_Se_(2‐x)_ nanosheets using the LLISA technique (one deposition) to form a complete, thin film. Figure 4a shows the photocurrent density (*J*_ph_) extracted from linear sweep voltammetry (LSV) measurements under intermittent 1‐sun illumination (presented in Figure S13) for iodide oxidation (inset Figure 4a). In the FTO/SnS_x_Se_(2‐x)_ device configuration, all materials containing some amount of sulfur show n‐type PEC activity. The pure SnSe_2_ (*x * =  0) nanosheets, however, do not exhibit any photoactivity on this substrate. The photocurrent onset potential exhibits a strong dependence on sulfur content, with a shift to lower applied bias observed as the sulfur content increases. Similarly, the saturation *J*_ph_ at 0.4 V vs. Pt pseudo‐reference electrode, summarized in Figure 4b, increases with the addition of sulfur and reaches a maximum around *x * =  1.6 of 43 µA cm^−2^ before decreasing again for the *x * =  2 sample. To ensure that these trends in PEC behavior stem from intrinsic nanosheet material performance and are not influenced by the contact to the FTO layer, we performed similar experiments employing SnO_2_ and TiO_2_ electron transport underlayers. LSV measurements under intermittent 1‐sun illumination for SnS_x_Se_(2‐x)_ nanosheets on SnO_2_ are presented in Figure S14 and summarized in Figure S15. Experiments with the TiO_2_ underlayer are presented in Figures S16 and S17. On these two underlayers, all samples show PEC activity for iodide oxidation, including the *x* = 0 sample that exhibits no activity on bare FTO. The trend in saturation *J*_ph_ over the range of nanosheet compositions remains the same on all substrates, with a maximum around *x* = 1.2 to 1.6 for S content. To study the effect of SnS_x_Se_(2‐x)_ nanosheet composition on the stability under PEC operation, devices (*x*  =  0.4 to 2) on bare FTO were tested in chronoamperometry (CA) measurements at an applied bias of 0.4 V vs. Pt. The corresponding CA curves are presented in Figure S18. All devices maintain photoelectrochemical activity over 60 min of continuous operation, independent of their composition.

Interestingly, the trend in photocurrent does not exactly follow electrical or optical trends, suggesting a more complex interplay of material properties. Previous work has shown that nanosheet length (*a*), thickness (*b*), and overall aspect ratio (*a/b*) are important factors when considering efficient photogenerated charge extraction [32]. Additionally, as the nanosheets lie flat along the substrate with the reaction taking place at the nanosheet basal planes, the desired direction of charge flow is perpendicular, or out‐of‐plane, rather than in‐plane. Moreover, it has been shown that the highest likelihood of charge recombination in 2D TMDs occurs at defect sites located at the edges of nanosheets [32, 37, 38], including for 2D SnS_2_ grown via chemical vapor deposition [39]. Thus, there is competition between the desired chemical reaction taking place at the nanosheet‐electrolyte interface and charge recombination at the nanosheet edge. As such, an important relationship exists between photocurrent and the nanosheet's conductivity out‐of‐plane and in‐plane.

We consider a simple analytical model (Equation 1, see derivation in Note S3) for the photocurrent density, *J*_ph_, of a single nanosheet that relates to charge generation, flake geometry, and charge mobility anisotropy. This model examines the probability of a photogenerated charge reaching the nanosheet‐electrolyte interface and participating in a chemical reaction versus annihilation at an edge site, where the majority of charge recombination takes place in untreated nanosheets [32, 37, 38, 39]. Recombination at edge defects is considered dominant and, as such, interfacial and trap‐assisted recombination are not considered here for simplicity. In (Equation 1), *G_0_* is the maximum charge generation rate, *µ_↑_* is the out‐of‐plane mobility, *µ_→_* is the in‐plane mobility, *e* is the elementary charge, *k* is the Boltzmann constant, *T* is the temperature, and *V_eff_* is the effective voltage drop across the flake thickness:

(1) $$J_{ph}=\frac{e^{2}bG_{0}}{6kT}\;{\left(\frac{a}{b}\right)}^{2}\frac{\mu_{↑}}{\mu_{\to }}V_{eff}$$

We expect that in an electrochemical cell, the applied potential, *ϕ*, is related to the voltage drop across the nanosheet, *V_eff_*, by: *ϕ = V_eff_ + A*, where *A* is an unknown constant related to interfacial contributions. Then, *dJ_ph_/dV* is equal to *dJ_ph_/dϕ*, where *ϕ* is the electrode potential, allowing for the linear region of the *J*_ph_‐*V* curve in Figure 4a to be expressed as:

(2) $$\frac{dJ_{ph}}{d\phi }=\frac{e^{2}bG_{0}}{6kT}\;{\left(\frac{a}{b}\right)}^{2}\frac{\mu_{↑}}{\mu_{\to }}$$

Indeed, Plainpan et al. have demonstrated *dJ/dV* analysis as an effective tool to evaluate photoelectrochemical systems using LSV, examining both the linear and plateau regions [40]. In contrast, and for simplicity, the model presented here only examines the linear region of the *J*_ph_‐*V* curve. Accordingly, experimental values for *dJ_ph_/dϕ* can be determined by extracting the slope (*dJ_ph_/dV*) of the linear (ohmic) region of the LSV curves shown in Figure 4a (as presented in Figure S19). Importantly, this trend closely mirrors that of the maximum *J*_ph_ observed in Figure 4b, suggesting that this simplistic model can be used as a proxy to investigate the factors influencing the maximum *J*_ph_. For a more detailed discussion of the assumptions made in this model see Note S3 in the Supporting Information. A bandgap‐dependent expression for the maximum charge generation rate, *G_0_*, derived assuming broadband, solar‐like illumination, is described in detail in Note S3. Notably, G_0_ only changes by a factor of three for the bandgap ranges considered here (1.67 to 2.54 eV). Moreover, while the bandgap of the SnS_x_Se_2‐x_ nanosheets increases linearly with increasing *x*, the observed maximum *J*_ph_ (and *dJ_ph_/dϕ*) do not follow this monotonic trend, further supporting that bandgap alone is not the determining factor for photocurrent in the alloyed nanosheets.

To fully explore this expression, flake dimensions *a* and *b* can be used from the previously discussed average nanosheet areas (Figure S6a) and average nanosheet thicknesses (Figure S6b), respectively. *E_g_* can be used from the Tauc plot analysis performed in Figure 3a. Thus, only the ratio of in‐plane and out‐of‐plane mobility (*µ_↑_/µ_→_*) of the SnS_x_Se_(2‐x)_ nanosheets remains an unknown.

To probe the nature of this relationship, vertical devices were fabricated to investigate out‐of‐plane conductivity. The devices were made by LLISA deposition onto indium‐doped tin‐oxide (ITO), which serves as the bottom electrode. Top electrodes were made by spray‐coating Ag nanosheets on top of the films, followed by annealing to sinter the Ag nanosheets and form conductive electrodes. A picture of the devices can be seen in Figure S20. Five LLISA depositions of SnS_x_Se_(2‐x)_ nanosheets were used to avoid device shorting, effectively forming a network of nanosheets. Typical *I*–*V* curves for the out‐of‐plane devices can be seen in Figure 4c. This behavior differs drastically from the in‐plane devices previously discussed. The current is relatively low for Se‐rich materials but increases for S content up to *x* = 1.6, after which it decreases for greater S content. SnSe_2_ could not be measured as the high Se content poisoned the Ag electrodes. Notably, all *I*–*V* curves appear to be linear due to the significantly reduced vertical channel length, which is equal to the network thickness. Based on the geometry of the devices, the out‐of‐plane conductivity for the networks can be estimated, as shown in Figure 4d. The ratio of out‐of‐plane to in‐plane conductivity (*σ_↑_/σ_→_)* for each alloy composition can then be examined using the extracted in‐plane and out‐of‐plane conductivities shown in Figures 3 and 4, respectively (ratio depicted in Figure S19b). Interestingly, *σ_↑_/σ_→_* follows a distinct composition dependence, initially increasing with S content up to *x* = 1.2 before slightly decreasing at *x* = 1.6, consistent with trends for the maximum *J*_ph_ and *dJ_ph_/dϕ*.

It is important to note that the model is for a single nanosheet and considers the attributes of a single nanosheet, while *σ_↑_/σ_→_* could only be calculated for a network of nanosheets, which includes additional contributions from nanosheet junctions [41, 42]. While junctions lower the absolute values, the trends should be comparable as the conductivity anisotropy mirrors mobility anisotropy under constant carrier density. Thus, to validate this model, *µ_↑_/µ_→_* (from Equation S4) is calculated to compare the theoretical nanosheet trend to the observed nanosheet network *σ_↑_/σ_→_* trend. The aforementioned experimental values can be used to calculate a theoretical *µ_↑_/µ_→_* as depicted in Figure S21. As expected, the spread of values for the calculated *µ_↑_/µ_→_* is lower than the experimentally observed *σ_↑_/σ_→_*, as a single nanosheet will not experience the same level of disorder caused by junctions in the nanosheet networks [41, 42]. Critically, the same trend is observed for the theoretical *µ_↑_/µ_→_* as with the experimentally determined *dJ_ph_/dϕ* and *σ_↑_/σ_→_* with values first increasing with S content, finding a maximum value at *x* = 1.6, and finally decreasing at higher S content.

These findings suggest that both the physical, electrical, and optical properties of these alloyed nanosheets are interconnected. Moreover, they are not wholly independent of one another, as changes in *a* or *b* would reasonably affect the in‐plane mobility, out‐of‐plane mobility, and *E_g_*. Importantly, the composition‐dependent PEC performance underscores the facility to tune the optoelectronic properties of SnS_x_Se_(2‐x)_ nanosheets and provides insight into the interconnectedness of material properties that enables tuning, which guides optimization in turn.

## 3 Conclusion

In summary, we developed a powder‐based, solution‐processable approach to synthesize composition‐tunable SnS_x_Se_(2‐x)_ nanosheets. By adjusting the feed ratio, we achieved the full ternary alloy series (*x* = 0 to *x* = 2) in 0.2 increments. Tauc analysis of nanosheet dispersions revealed a wide range of composition‐dependent optical bandgaps (1.67 – 2.46 eV). Using scalable printing methods, we fabricated thin films to investigate their electronic and optoelectronic properties. In‐plane devices exhibited n‐type transport, with Se‐rich alloys being more conductive and S‐rich alloys more insulating. Out‐of‐plane devices showed a distinct nonmonotonic trend, with conductivity increasing up to S content with *x* = 1.6 before decreasing. To probe the combined impact of these behaviors, we employed PEC devices, which revealed that optoelectronic performance does not follow any single material parameter. Instead, it emerges from the interplay of nanosheet geometry, mobility anisotropy, and the effective voltage drop across the flake. This composition‐property relationship highlights the ability to tune SnS_x_Se_(2‐x)_ nanosheets for optimized optoelectronic performance, providing guiding principles for future materials engineering and device design across a range of applications. For example, looking ahead to printed electronics based on SnS_x_Se_(2‐x)_ nanosheets, an optimal alloy composition is in the *x* = 0.8 to 1.2 range. However, for PEC systems, an alloy composition in the *x* = 1.2 to 1.6 range achieves optimized performance. Altogether, this work opens avenues toward a versatile new platform for the scalable production of highly tunable 2D materials.

## 4 Experimental Section/Methods

### 4.1 Materials

Sn powder (99.995%) and Se powder (99.999%) were purchased from abcr. SnS_2_ powder (99.5%) was purchased from eNovation Chemicals LLC. S powder (99.98%) and tetrabutylammonium hexafluorophosphate (98%) were purchased from Sigma Aldrich. LiI was purchased from Fluorochem. Tetraheptylammonium bromide (99%) was purchased from Acros Organics. N‐methylpyrrolidone (99%) and EtOH (99.8%) were purchased from Thermo Scientific. Acetonitrile (UPLC grade) was purchased from Carlo Erba Reagents. Hexane was purchased from EMSURE. All chemicals were used without further purification.

### 4.2 Bulk SnSe_2_ Preparation

Bulk SnSe_2_ powder was prepared following an adapted literature protocol [19]. Briefly, Sn and Se powder (1:2 molar ratio) were finely ground together, loaded into a fused quartz tube, and vacuum‐sealed. The mixed powders were heated from room temperature to 750°C over 12 h, held at 750°C for 10 h, and then allowed to cool naturally. The resulting sintered material was finely ground into a powder with a pestle and mortar and used like this for further experiments.

### 4.3 Bulk Material Alloy Pellet Preparation

Bulk material alloy pellet preparation was performed following an adapted literature protocol [10]. Briefly, SnS_2_, SnSe_2_, or a desired ratio of the two was mixed and finely ground together with a pestle and mortar. 400 mg of powder, or powder mixture for the alloys, were pressed with up to 12 t in a 12.5 mm diameter die using a manual 25 t hydraulic press (Hercules, PD Instruments) and held for 10 s. Up to three pressed pellets of the same composition were vacuum sealed in a fused quartz glass tube together with powder (100 mg) of S, Se, or a mixture of the two of the same molar ratio as SnS_2_ and SnSe_2_ in the pellets. It was ensured that the pellets were not touching inside the sealed tube to avoid fusing. SnS_x_Se_(2‐x)_ pellets containing selenium (0% to 90% S content) were heated from room temperature to 600°C over 6 h, held at 600°C for 48 h, and then allowed to cool naturally. SnS_2_ pellets were heated from room temperature to 650°C over 6 h, held at 650°C for 12 h, and then allowed to cool naturally.

### 4.4 Electrochemical Pellet Intercalation and Exfoliation

Electrochemical pellet intercalation was done following a literature protocol [10, 43]. An annealed pellet was clipped with a metal alligator clip and placed in a 50 mL glass beaker with a glassy carbon counter electrode. Both electrodes were connected to a potentiostat with the pellet as the working electrode. A solution (5 mg mL^−1^) of tetraheptylammonium bromide (THAB) in acetonitrile was added until the pellet was submerged without contact between the liquid and the alligator clip. A voltage of 10 V was applied, with the pellet as the cathode, for 5–72 h, depending on the pellet composition. Pellets with higher sulfur content need longer intercalation times. The end of the intercalation process can be determined by the amount of material that has sloughed off the pellet working electrode, where longer intercalation times lead to higher collected material amounts and thus greater intercalated material yield. The powder sloughed off the pellet during the intercalation was collected, washed with ethanol, resuspended in N‐methylpyrrolidone (NMP), and subjected to 2 h of bath sonication. Finally, the resulting dispersion was centrifuged (30 min, 120 rcf) and the top 8 mL of supernatant were collected, containing the exfoliated pTMD nanosheets.

### 4.5 TiO_2_ and SnO_2_ Underlayer Preparation

FTO‐coated glass substrates (Solaronix SA) were subsequently cleaned with acetone, DI water, and isopropanol (IPA) for 20 min under sonication, followed by drying. Before deposition of the following layers, the substrates were subjected to UV ozone cleaning (UV Ozone Cleaner, Ossila Ltd.) for 15 min. SnO_2_ was deposited by spin‐coating a precursor solution of colloidal SnO_2_ nanoparticles (3 wt.% in H_2_O) onto the FTO substrates (3000 rpm, 2500 rpm s^−1^, 30 s) and subsequent annealing at 160°C for 1 h. TiO_2_ was deposited on FTO substrates (heated at 450°C) by spray pyrolysis of a precursor solution of titanium diisopropoxide bis(acetylacetonate) (75 wt.% in isopropanol) in EtOH, using an oxygen carrier gas. Subsequently, the substrates were annealed at 450°C for 30 min. Layer thicknesses of TiO_2_ and SnO_2_ were determined to be 10–30 nm and 10–20 nm by profilometry, respectively.

### 4.6 Thin Film Formation

Nanosheet thin films were made via a previously‐described liquid‐liquid interfacial self‐assembly (LLISA) approach [10, 43]. Briefly, nanosheets dispersed in NMP were slowly injected into an H_2_O/hexane interface until complete film formation. For SnS_2_ nanosheets, the aqueous layer was exchanged for an aqueous solution of HCl (0.1 m). After aspirating the hexane layer, pre‐positioned substrates (cleaned, bare FTO, TiO_2_‐coated FTO, or SnO_2_‐coated FTO) were lifted out of the aqueous layer to transfer the nanosheet film onto the substrate. After drying in air, the films were annealed at 200°C for 120 min in a vacuum oven to remove excess solvent and ensure good contact between the nanosheets and the substrate.

For in‐plane and out‐of‐plane electrical devices, nanosheets dispersed in IPA were slowly injected into an H_2_O/hexane interface until complete film formation. After film formation, pre‐positioned substrates (pre‐patterned Fraunhofer chips, pre‐patterned ITO) were lifted through the liquid interface to transfer the nanosheet film onto the substrate. Three and five subsequent layers were deposited for in‐plane and out‐of‐plane electrical devices, respectively, with air‐drying and mild heating (100°C) in between depositions. After drying in air, the films were annealed at 200°C for 120 min in a vacuum oven to remove excess solvent and ensure good contact between the nanosheets and the substrate.

For out‐of‐plane devices, top electrodes were deposited by spraying silver nanosheets dispersed in DI H_2_O (10 mg mL^−1^) through a mask to give electrode areas of 4 mm^2^ and a thickness of ∼1.5 µm. The spray deposition hot plate is set to 150°C. After air drying, the devices are annealed under vacuum at 200°C to sinter the Ag nanosheets into a continuous electrode. Ag nanosheets were chosen to avoid shorting caused by nanoparticle penetration during deposition.

### 4.7 Characterization

*UV–vis* *spectra* were acquired using a Shimadzu UV‐3600 spectrometer from 900 to 260 nm, with an integrating sphere, a step size of 1 nm, and a slit width of 5 nm. Measurements in dispersions in NMP were taken directly in transmission mode using a quartz cuvette. Nanosheet dispersions were diluted in NMP, and NMP was used as a blank. Absorbance was calculated with

(3) Absorbance=2−log10(%T)

where *%T* is the percent transmission

*Raman spectra* were obtained on sintered pellets or single nanosheet‐layer films (one LLISA deposition) on glass with a Horiba Xplora Plus Raman microscope with 532 nm radiation. Raman spectra were acquired from 50 – 1000 cm^−1^ using a 100*x* objective, slit of 200 µm, hole of 500 µm, 10% filter, 2400 gr/mm grating, 8 s of acquisition, and 6 accumulations.

*X‐ray diffraction* patterns were obtained in Bragg‐Brentano geometry using monochromatic Cu Kα1 radiation on a Bruker D8 Vario equipped with a LynxEye XE detector. For the measurements, pellets were ground into fine powder using a pestle and mortar and placed directly on the sample holder.

*SEM* images were acquired using a Zeiss Merlin operated at 2 kV with a probe current of 100 pA and a working distance of about 2.7 mm using an in‐lens detector. Pellets were electrically grounded using a copper clip.

*TEM, HAADF STEM*, and *STEM EDX* images were acquired on an FEI Talos F200s microscope at 200 kV. For HAADF STEM images, a probe current of 100 pA, a camera length of 125 mm (collection angle > 75 mrad), and a well of 1–5 µs was used. Samples for TEM and STEM were prepared by drop‐casting a dilute dispersion of nanosheets in IPA onto TEM grids (∼3 nm amorphous carbon on 400 µm copper mesh, Ted Pella Inc., USA), followed by drying in vacuum at 200°C for at least 2 h.

*In‐plane electrical* devices were made by performing three LLISA depositions (as described above) onto n‐doped silicon substrates with 230 nm SiO_2_ gate oxide and ITO/Au source‐drain contacts (Gen 4, W = 10 mm, OFET Test Chips, IPMS Fraunhofer, Dresden, Germany). Electronic testing was performed using a Janis ST‐500 Probe Station (Lake Shore Cryotronics Ltd., OH, USA) in conjunction with a Keithley 4200A‐SCS Parameter Analyzer (Keithley Instruments, Ohio, US).

*Out‐of‐plane electrical* devices were made by performing five LLISA depositions onto ITO‐coated glass with Ag top electrodes. Electronic testing was performed using a Keithley 2612A with a Suss probe station.

*Atomic force microscopy* was carried out using a Cypher S (Asylum Research) equipped with a silicon cantilever (Olympus AC160TS‐R3) in alternating current (AC) tapping mode. Statistical thickness distributions were analyzed using Gwyddion, with a minimum of 125 nanosheets being measured. Flake size distributions over at least 400 nanosheets were analyzed using ImageJ.

For *PEC measurements*, single nanosheet‐layer films (one LLISA deposition) deposited on FTO, SnO_2_‐coated FTO, or TiO_2_‐coated FTO were used as photoelectrodes directly. Linear sweep voltammetry (LSV) and chronoamperometry (CA) measurements were performed using a three‐electrode system with the nanosheet thin film as the working electrode, a carbon counter electrode, and a Pt pseudoreference electrode. The active area of the electrode was 0.245 cm^2^. The electrolyte was 50 mM LiI and 25 mM tetrabutylammonium hexafluorophosphate in acetonitrile. For LSV measurements, the applied voltage was swept from ‐0.3 V to +0.5 V (vs. Pt) at a scan rate of 10 mV s^−1^. A 450 W Xe light source with a KG2 filter was used and calibrated to 1‐sun intensity. Illumination was intermittent during LSV measurement, with 2 s of illumination followed by 1 s without. CA measurements were performed at an applied voltage of 0.4 V vs. Pt for a duration of 60 min. The samples were illuminated continuously for 10 min, followed by 10 s of operation in the dark. All samples were measured with the FTO‐coated glass substrate side toward the light source (back‐illumination).

*Statistical Analysis* was performed for nanosheet morphological characterization, photocurrent analysis, in‐plane electrical measurements, out‐of‐plane electrical measurements, and modelling of *J*_ph_ based on empirical values Table 1.

**TABLE 1:** Summary of statistical analysis information.

Measurement	Figure	Data Presentation	Sample Size	Evaluation of Outliers
In‐Plane Electrical Measurements	Figure 3c, Figure S12	Mean + Standard Deviation	4 samples	Samples more than one order of magnitude different from the sample mean were considered outliers
Photocurrent	Figure 4b, Figures S15b and S17b	Mean + Standard Deviation	6 Samples	N/A
Out‐of‐Plane Electrical Measurements	Figure 4d,	Mean + Standard Deviation	1‐4 samples	N/A
Nanosheet Area Analysis	Figure 2g, Figure S6a,	Mean + Standard Error	Minimum 400 nanosheets	N/A
Nanosheet Thickness Analysis	Figure S6b	Mean + Standard Error	Minimum 125 nanosheets	N/A

## Funding

R.A.W. acknowledges the SNSF Postdoc.Mobility fund for financial support (project P500PN_217967). T.C. has received funding from the European Union's Horizon Europe research and innovation programme, Grant agreement No. 101129613 (HYPERSONIC). G.G. and L.D.A.S. received funding from the Netherlands Organization for Scientific Research (NWO) in the framework of the Materials for Sustainability and from the Ministry of Economic Affairs in the framework of the PPP allowance.

## Conflicts of Interest

The authors declare no conflicts of interest.

## Supporting information




**Supporting File**: smll72204‐sup‐0001‐SuppMat.pdf

## Data Availability

The data that support the findings of this study are available from the corresponding author upon reasonable request.
