# Supplementary material for: Composition‐Dependent Wide‐Range Tunability of Optical and Electronic Properties in SnSxSe(2‐x) Alloy Nanosheets
Source: Small. 2025 Dec 31;22(11):e12066. doi: 10.1002/smll.202512066 (PMC12921546; doi:10.1002/smll.202512066)
Supplement: Supplementary file 1 — Supporting File: smll72204‐sup‐0001‐SuppMat.pdf [file SMLL-22-e12066-s001.pdf]

Supporting Information

Composition-Dependent Wide-Range Tunability of Optical and Electronic Properties in Printable SnS_xSe_{2-x} Alloy Nanosheets

*Nicolas J. Diercks, Rebekah A. Wells, Shixin Liu, Tian Carey, Jack Doran, Joseph Neilson, YeonJu Kim, Jun-Ho Yum, Goutam Ghosh, Hannah Johnson, Laurens D. A. Siebbeles, Jonathan N. Coleman, and Kevin Sivula**

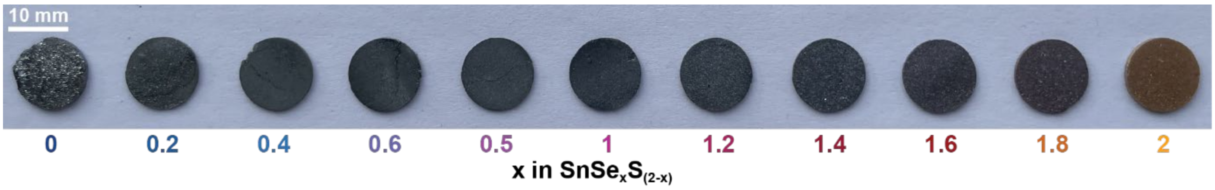

Figure S1. Photograph of sintered $SnS_xSe_{(2-x)}$ pellets with varying sulfur content from $x = 0$ to 2.

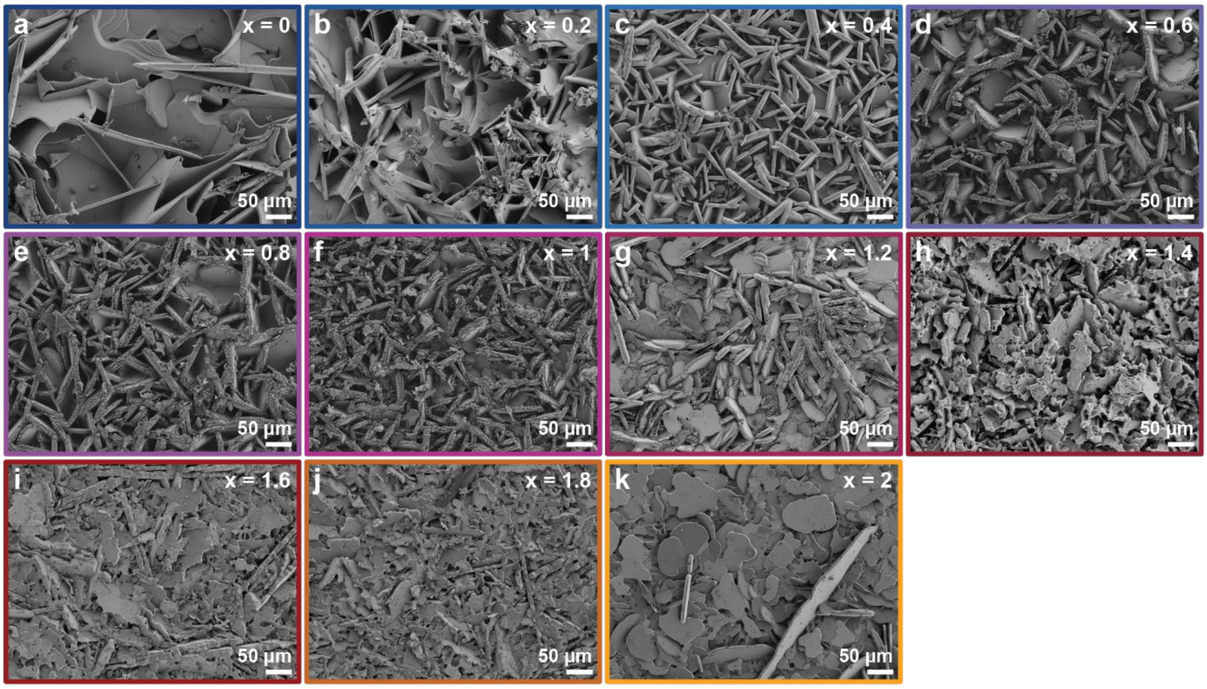

Figure S2. SEM images of the surface of sintered $SnS_xSe_{(2-x)}$ pellets with varying sulfur content $x = 0$ to 2.

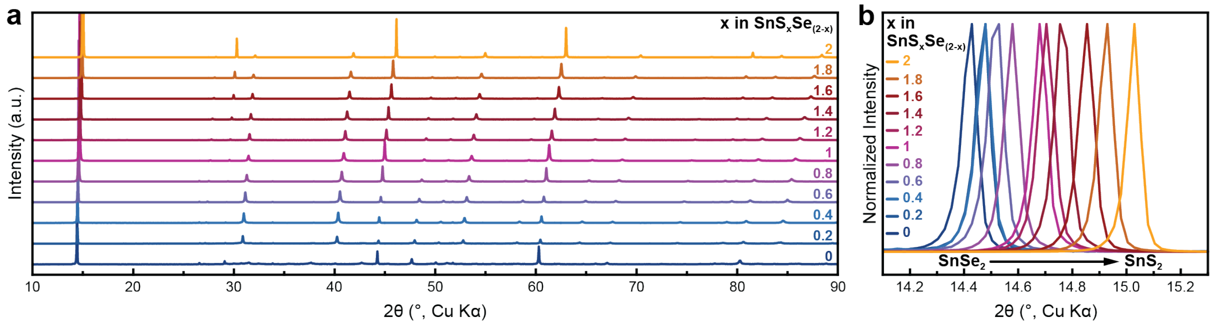

Figure S3. (a) Stacked PXRD pattern acquired from sintered, ground $SnS_xSe_{(2-x)}$ pellets with varying sulfur content (x = 0 to 2). (b) Normalized (001) reflection of the spectra in (a).

Supplementary Note 1: **Figure S3** shows the PXRD of ground, sintered pellets with different S to Se feed ratios. As visible in **Figure S3a**, the incorporation of S to replace Se in the crystal structure leads to a gradual shift of the lattice reflections to higher degrees, which is in line with a decrease in lattice constants with the incorporation of the smaller S atoms. The PXRD spectra show no further phases than the expected CdI_2-type structures of $SnSe_2$, SnS_2, or the alloy-compositions ($SnS_xSe_{(2-x)}$) in between. **Figure S3b** shows the normalized (001) reflection peak, which further emphasizes the gradual shift. The (001) reflection of $SnSe_2$ (x = 0) is found at 2θ = 14.43° with the peak position gradually shifting (with increasing sulfur content) to 15.03° for SnS_2 (x = 2).

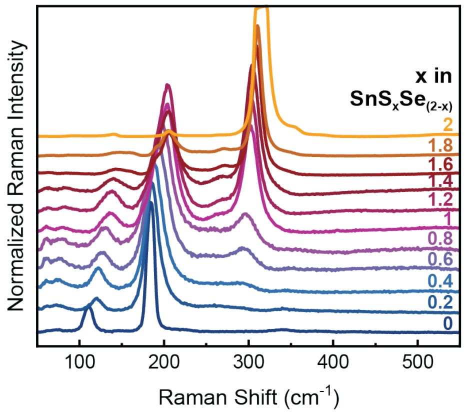

Figure S4. Stacked Raman spectra of sintered $SnS_xSe_{(2-x)}$ pellets with varying sulfur content (x = 0 to 2).

Supplementary Note 2: **Figure S4** shows the normalized, stacked Raman spectra acquired on sintered pellets with different S to Se feed ratios. For pure $SnSe_2$ (x = 0), two prominent peaks are visible. The signals at 110.5 cm^{-1} and 184.4 cm^{-1} can be assigned to the $SnSe_2$ E_g and A_{1g} modes, respectively.[1] With increasing sulfur content, the intensity of the two modes slowly decreases while a peak at 292.1 cm^{-1} starts emerging for S content of x = 0.4. This peak can be assigned to the SnS_2 A_{1g} mode.[1] With changing intensity ratios of the $SnSe_2$ and SnS_2 peaks, the peak positions also shift to higher wavenumbers. The $SnSe_2$ E_g and A_{1g} peak positions

gradually shift from 110.5 cm^{-1} and 184.4 cm^{-1} ($x = 0$) to 147.6 cm^{-1} and 205.5 cm^{-1} ($\underline{x} = 1.8$), respectively. The $SnSe_2$ A_{1g} mode starts overlapping with the weak SnS_2 E_g mode at higher S concentrations until only the signal from the SnS_2 E_g mode[1] is visible for the pure SnS_2 ($x = 2$) sample at 205.5 cm^{-1}. The emerging SnS_2 A_{1g} mode gradually shifts from its position at 292.1 cm^{-1} for the 20% S sample to 314.8 cm^{-1} for SnS_2 ($x = 2$). The unique Raman signals for the different compositions, composed of shifted signals of the pure materials, rather than the appearance of pure SnS_2 and $SnSe_2$ signals next to each other, further prove the formation of $SnS_xSe_{(2-x)}$ alloys in the bulk, with the composition dependent on the feed concentration of SnS_2 and $SnSe_2$ before pellet sintering.

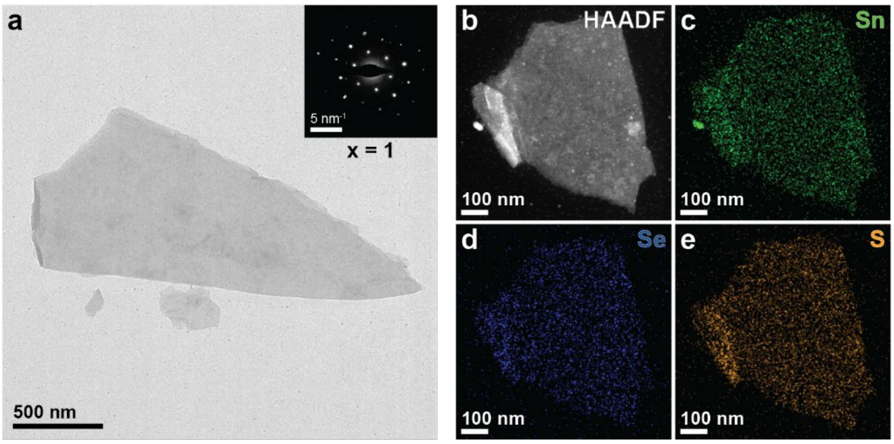

Figure S5. TEM and STEM of SnSSe nanosheets ($x = 1$). (a) Bright-field TEM image of a SnSSe nanosheet. The inset shows the SAED pattern. (b) STEM HAADF image of a SnSSe nanosheet. (c), (d), and (e) show the elemental distribution of Sn, Se, and S, respectively, measured by STEM EDX.

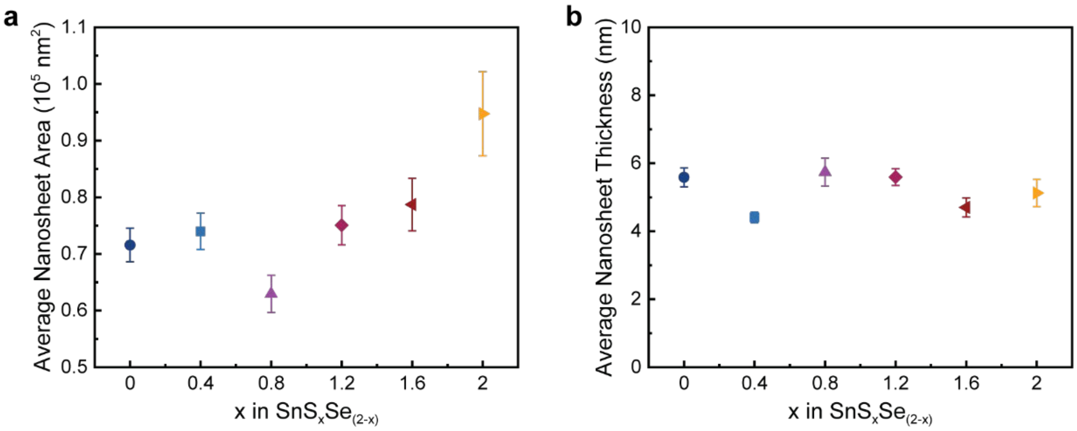

Figure S6. Morphological analysis of $SnS_xSe_{(2-x)}$ nanosheets depending on the composition ($x = 0$ to 2). (a) Extracted nanosheet areas (average + standard error of at least 400 measured nanosheets). (b) Extracted nanosheet thicknesses (average + standard error of at least 125 measured nanosheets).

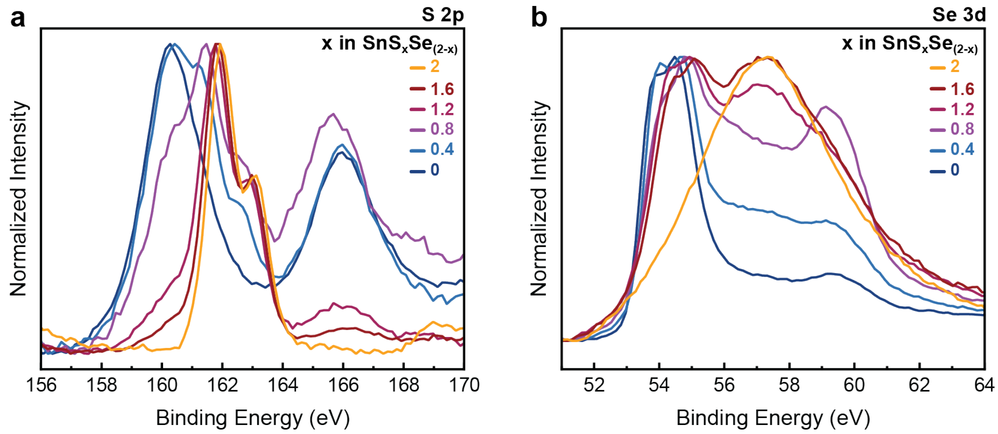

Figure S7. Normalized XPS spectra of (a) the S 2p and (b) the Se 3d peaks of $SnS_xSe_{(2-x)}$ nanosheet thin films with varying sulfur content (x = 0 to 2).

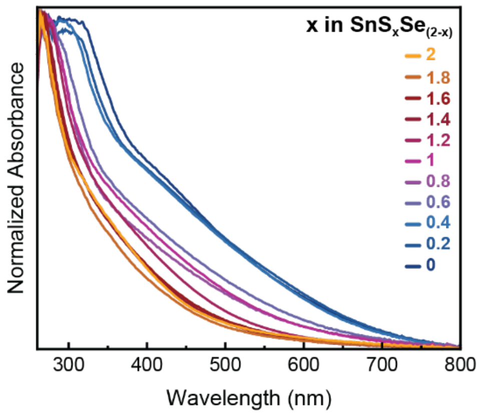

Figure S8. Normalized UV-vis spectra of $SnS_xSe_{(2-x)}$ nanosheet dispersions in NMP with varying sulfur content (x = 0 to 2).

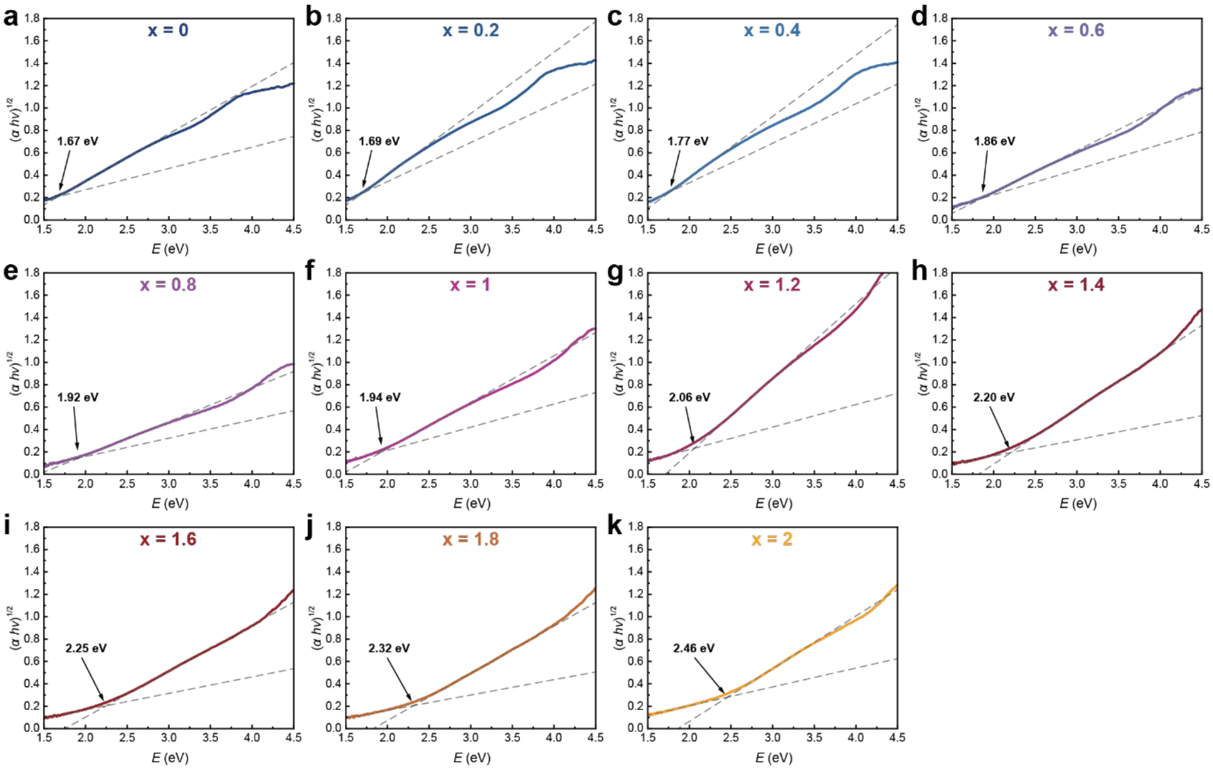

Figure S9. Tauc analysis of $SnS_xSe_{(2-x)}$ nanosheet dispersions in NMP with varying sulfur content (x = 0 to 2). Nanosheets were prepared with (a) x = 0, (b) x = 0.2, (c) x = 0.4, (d) x = 0.6, (e) x = 0.8, (f) x = 1, (g) x = 1.2, (h) x = 1.4, (i) x = 1.6, (j) x = 1.8, and (k) x = 2.

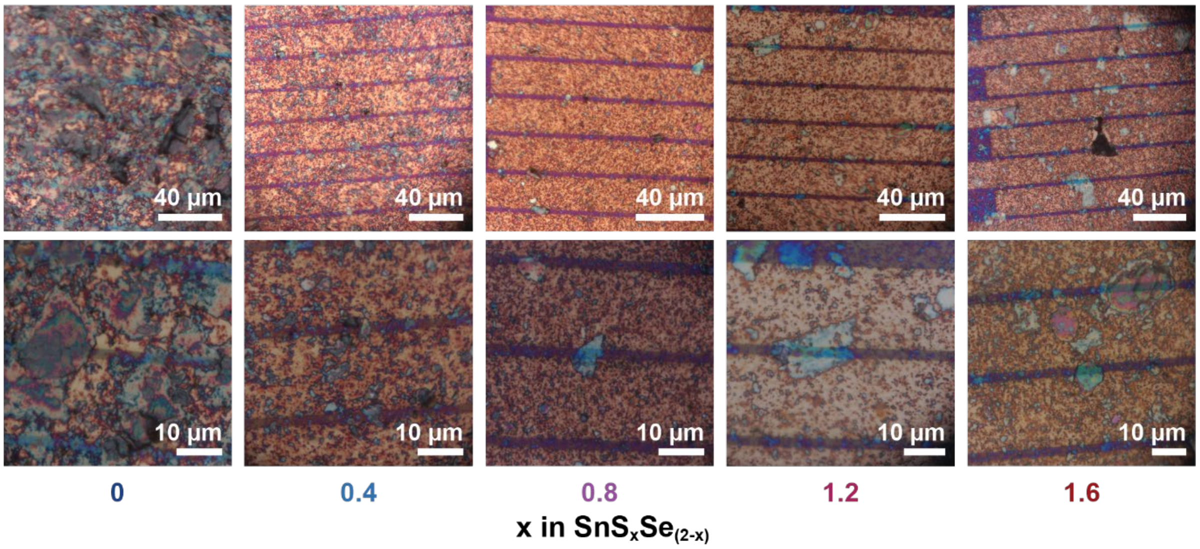

Figure S10. Optical microscopy images of 2.5 µm channel devices used for in-plane electrical testing. Nanosheet films were deposited on pre-patterned Fraunhofer (gen 4) electronic testing chips using the LLISA printing technique (three depositions).

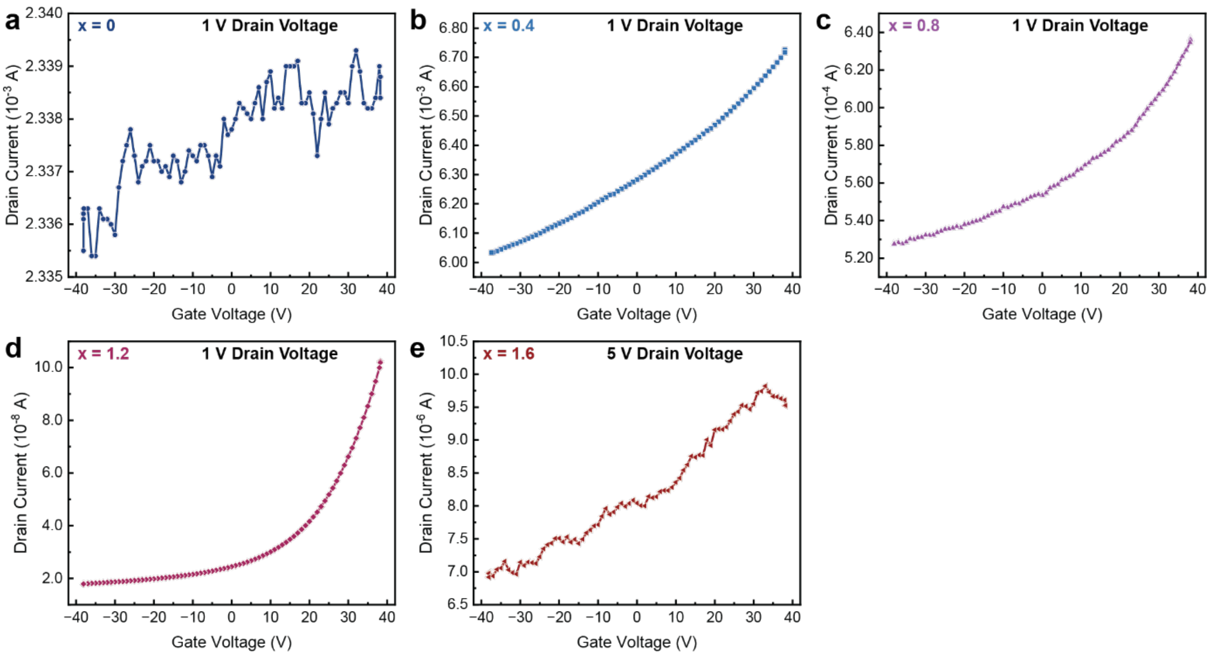

Figure S11. Typical transfer curves for in-plane electrical devices of $SnS_xSe_{(2-x)}$ nanosheets deposited on Gen 4 Fraunhofer chips for (a) $x = 0$, (b) $x = 0.4$, (c) $x = 0.8$, (d) $x = 1.2$, and (e) $x = 1.6$. The channel length and width are 2.5 µm and 10 mm, respectively. Transfer curves for drain voltages = 1 – 5 V could not be reliably obtained for SnS_2 ($x = 2$) due to the high resistivity of the material. Indeed, the transfer curve obtained for $x = 1.6$ is already at the lower limit of detection and therefore is not considered in subsequent mobility and charge carrier concentration calculations.

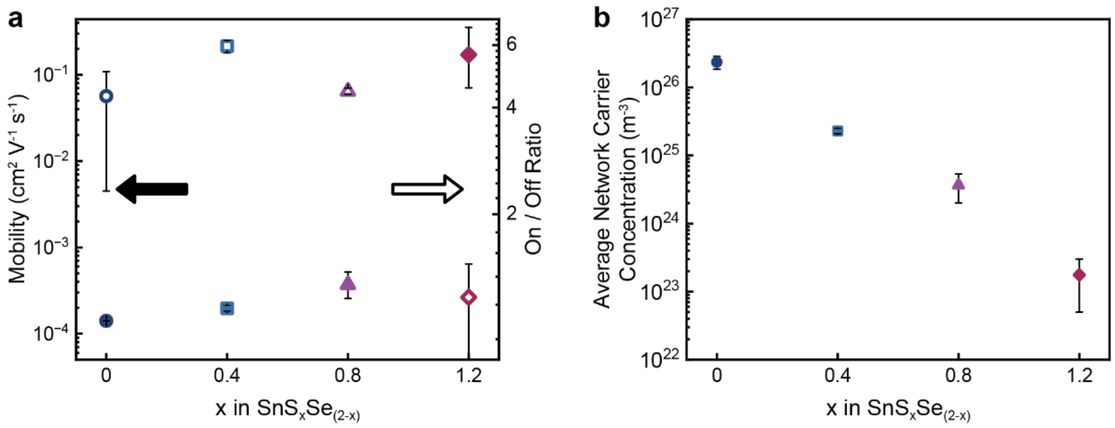

Figure S12. (a) Calculated field-effect mobilities (closed icons) and on/off ratios (open icons) for in-plane $SnS_xSe_{(2-x)}$ devices for $x = 0$ to 1.2. Average of four devices. (b) Average network carrier concentration for $x = 0$ to 1.2. The channel length and width are 2.5 µm and 10 mm, respectively. Error bars are for standard deviation from mean.

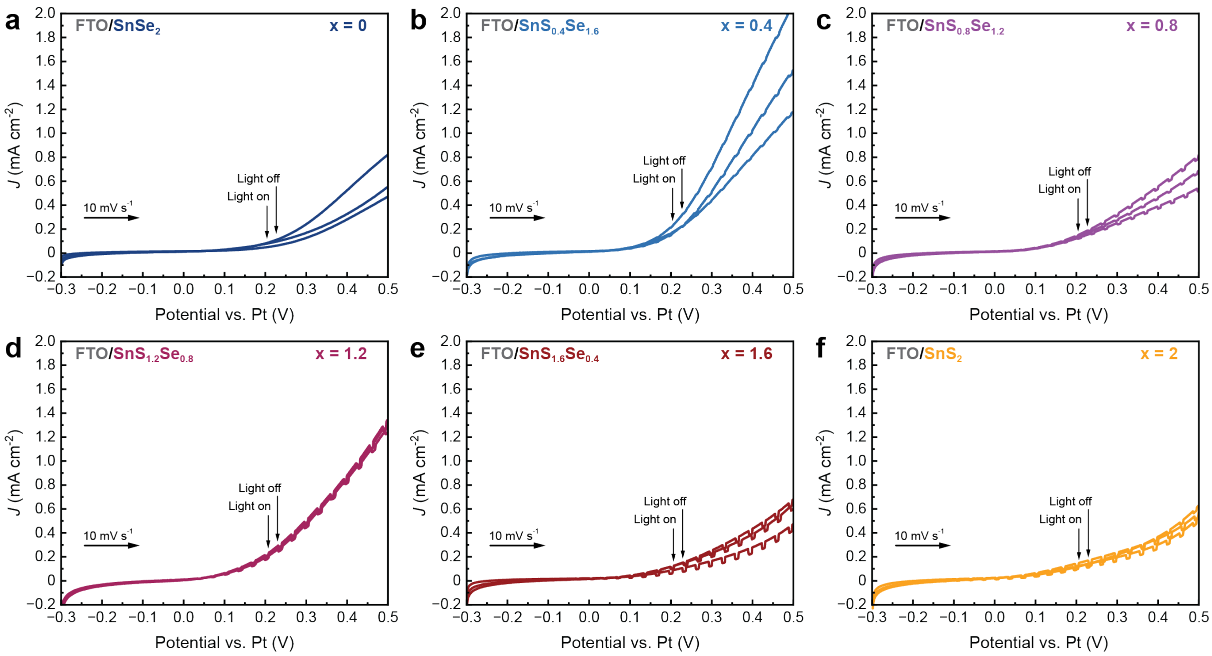

Figure S13. LSV curves of FTO/$SnS_xSe_{(2-x)}$ nanosheet devices under intermittent 1-sun illumination for iodide oxidation in acetonitrile. Sulfur content in $SnS_xSe_{(2-x)}$ nanosheets was (a) $x = 0$, (b) $x = 0.4$, (c) $x = 0.8$, (d) $x = 1.2$, (e) $x = 1.6$, and (f) $x = 2$.

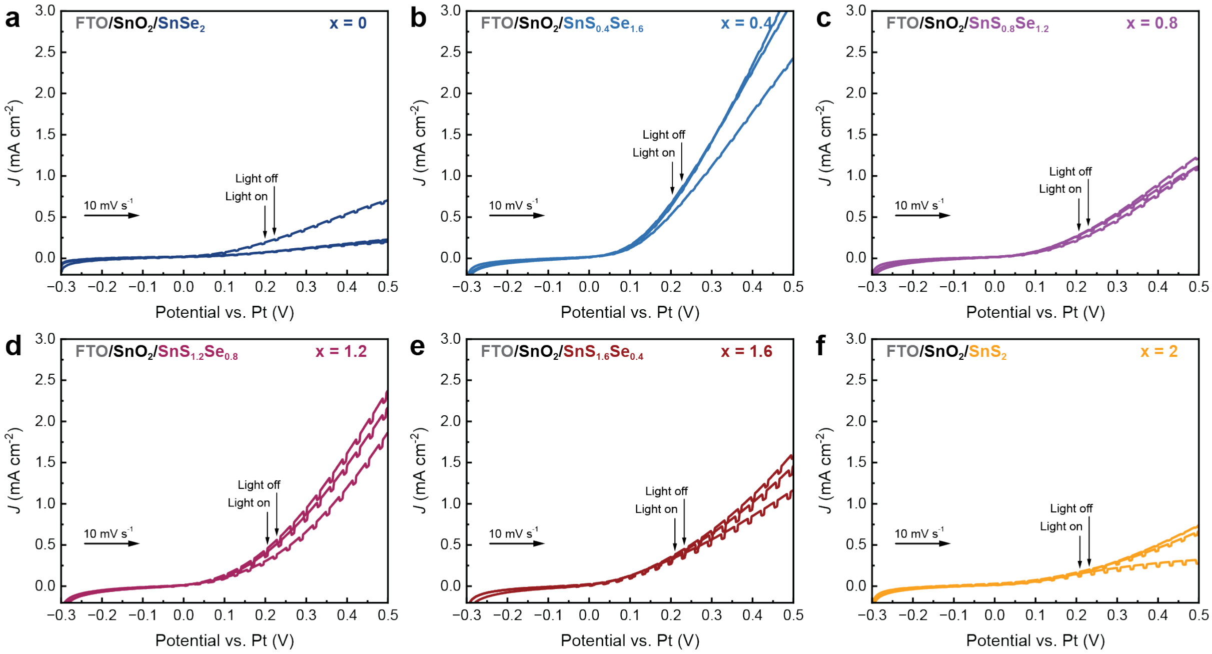

Figure S14. LSV curves of FTO/SnO_2/$SnS_xSe_{(2-x)}$ nanosheet devices under intermittent 1-sun illumination for iodide oxidation in acetonitrile. Sulfur content in $SnS_xSe_{(2-x)}$ nanosheets was (a) $x = 0$, (b) $x = 0.4$, (c) $x = 0.8$, (d) $x = 1.2$, (e) $x = 1.6$, and (f) $x = 2$.

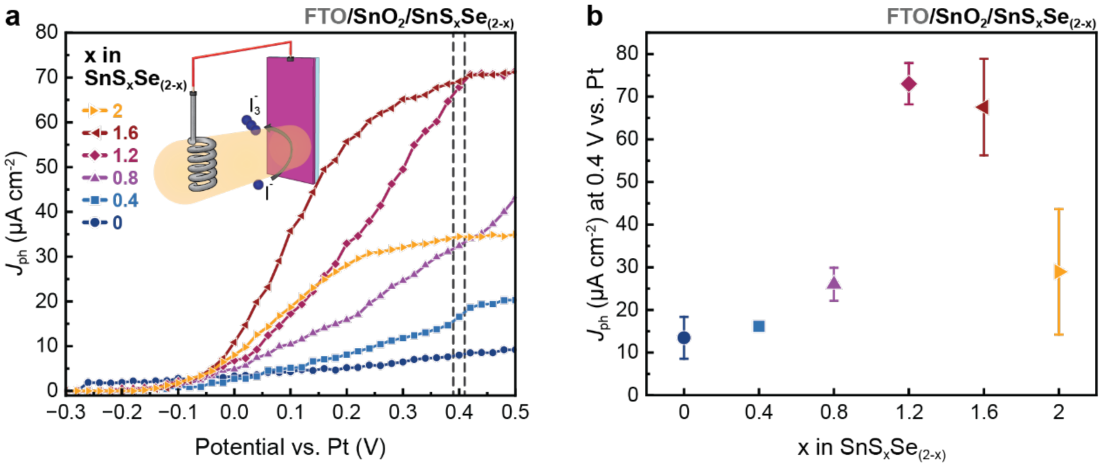

Figure S15. PEC performance summary of FTO/SnO_2/$SnS_xSe_{(2-x)}$ nanosheet devices under intermittent 1-sun illumination for iodide oxidation in acetonitrile. (a) Extracted photocurrent from LSV curves under intermittent illumination of example devices. (b) Comparison of photocurrent at 0.4 V vs. Pt for the varying compositions. Error bars present the standard deviation from mean.

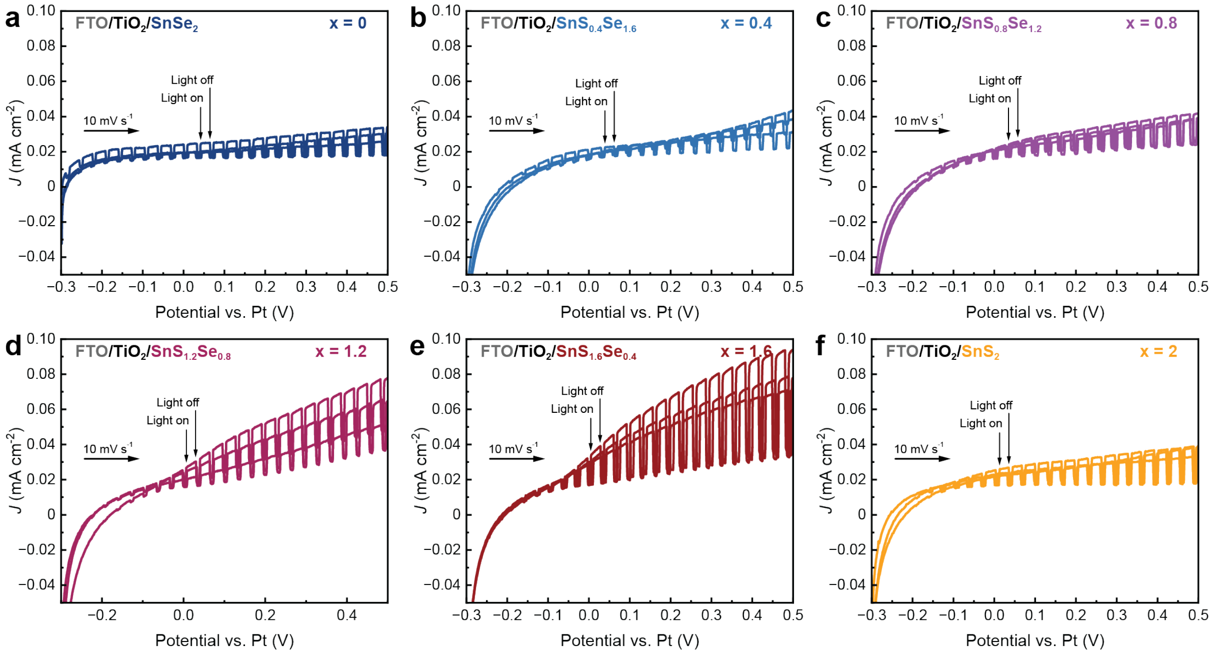

Figure S16. LSV curves of FTO/TiO_2/$SnS_xSe_{(2-x)}$ nanosheet devices under intermittent 1-sun illumination for iodide oxidation in acetonitrile. Sulfur content in $SnS_xSe_{(2-x)}$ nanosheets was (a) $x = 0$, (b) $x = 0.4$, (c) $x = 0.8$, (d) $x = 1.2$, (e) $x = 1.6$, and (f) $x = 2$.

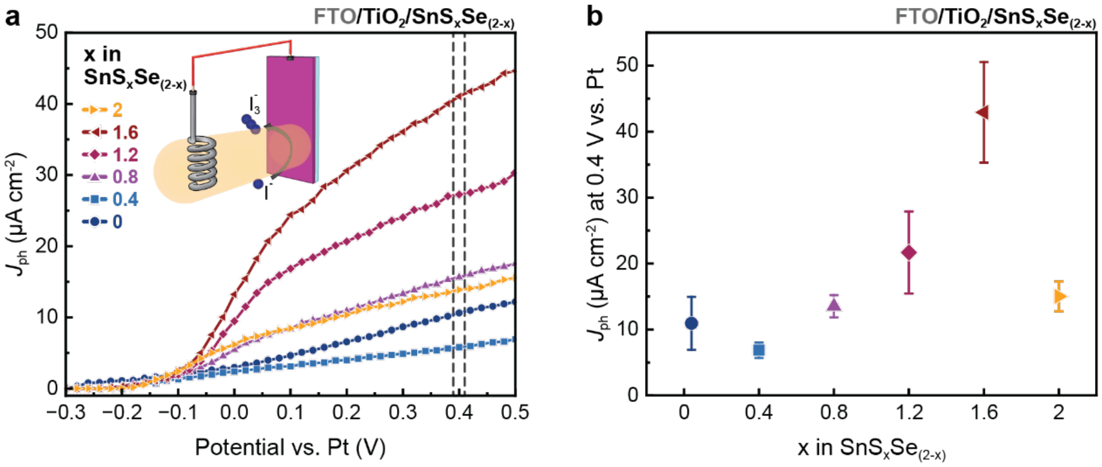

Figure S17. PEC performance summary of FTO/TiO_2/$SnS_xSe_{(2-x)}$ nanosheet devices under intermittent 1-sun illumination for iodide oxidation in acetonitrile. (a) Extracted photocurrent from LSV curves under intermittent illumination of example devices. (b) Comparison of photocurrent at 0.4 V vs. Pt for the varying compositions. Error bars present the standard deviation from the mean.

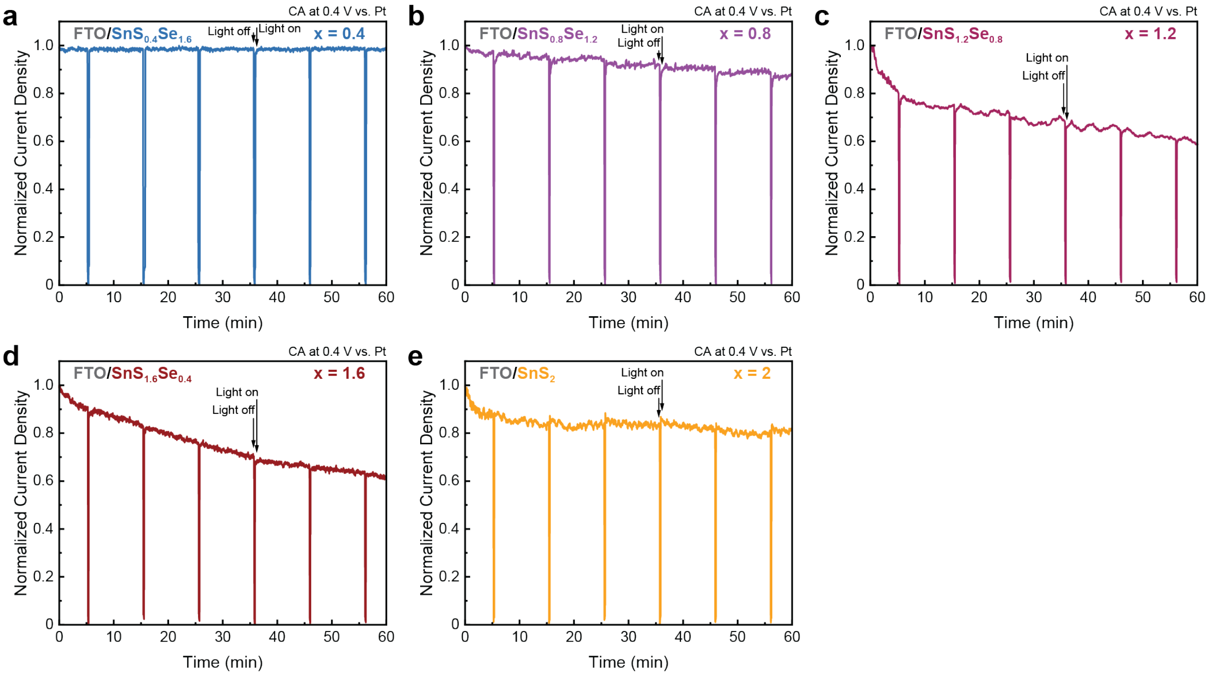

Figure S18. Normalized chronoamperometry (CA) curves of FTO/$SnS_xSe_{(2-x)}$ nanosheet devices for $x = 0.4$ to 2 under continuous 1-sun illumination for photoelectrochemical iodide oxidation in acetonitrile. CA measurements were performed at an applied bias of 0.4 V vs. Pt.

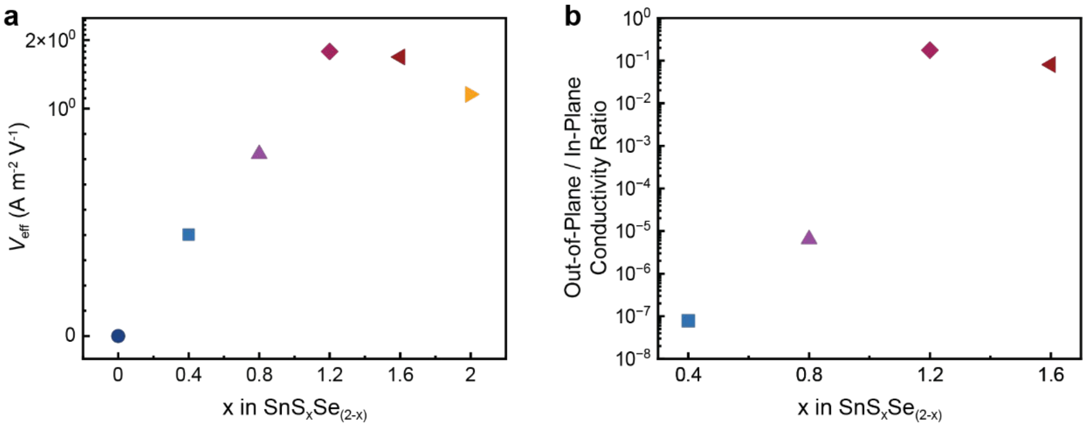

Figure S19. (a) *dJ*/*dV* extracted from the linear region of the LSV curves shown in Figure 4 of the main text for $x = 0$ to 2. (b) Ratio of out-of-plane to in-plane conductivity for $x = 0.4$ to 1.6. In-plane conductivity could not be determined for $x = 2$ due to material resistivity. Out-of-plane conductivity could not be determined for $x = 0$ as the Se content poisoned the Ag electrodes during sintering.

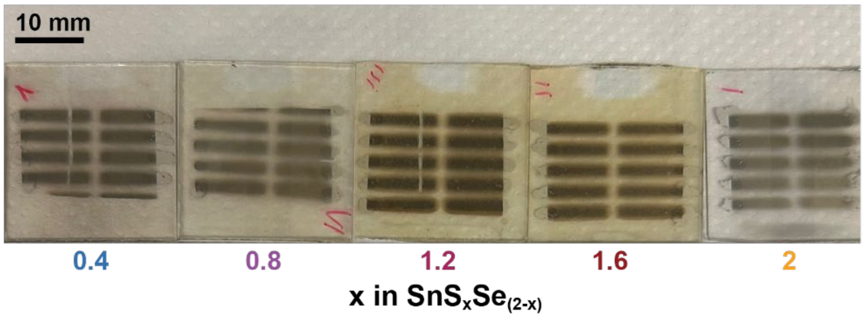

Figure S20. Image of vertical devices for out-of-plane electrical devices. Five layers of $SnS_xSe_{(2-x)}$ nanosheets deposited on ITO-patterned (middle strip) glass with sintered Ag nanosheet top electrodes. Ag contacts could not be made for $x = 0$ as the Se content poisoned the Ag electrodes during sintering.

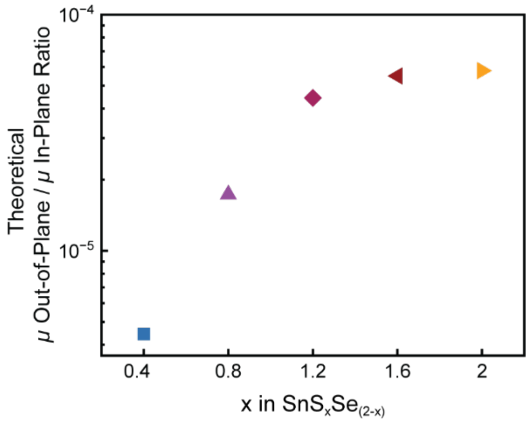

Figure S21. Theoretical $\mu_{\uparrow}/\mu_{\rightarrow}$ calculated from **Equation S4** in the Supplementary Information.

Supplementary Note 3: Deriving an expression for bandgap-dependent photocurrent

3.1 Finding the boundary of the active region where photogenerated charge will reach the nanosheet-electrolyte interface.

Consider a disc-like nanosheet (radius a, thickness b) on a back electrode with the top immersed in electrolyte. This is shown in **Figure S22** with cylindrical polar coordinates included. The system is illuminated from the backside through the (transparent) electrode. A potential difference is applied between the electrode and the electrolyte.

Consider an exciton generated at the point (r, z). Such excitons are only of interest in this context if they split into an electron (e)-hole (h) pair (facilitated by the applied potential). We make the simplification that the e-h pair will always have only two possible endpoints (see section on model assumption below). One possibility is that the electron (in our case) moves to the electrode with the hole moving toward the nanosheet surface/electrolyte interface, roughly along the path of the red dashed arrow (**Figure S22**). This process will be driven by the applied potential. We assume that once the hole reaches the nanosheet-electrolyte interface, it will immediately participate in an electrochemical reaction. Alternatively, the pair can be annihilated at the nanosheet edge as demonstrated by our previous works,[2-4] as well as work on SnS_2 nanosheet grown *via* chemical vapor deposition.[5] We might imagine that the hole will diffuse toward the edge of the nanosheet approximately along the path given by the blue dashed arrow. We assume that once it reaches the edge, it will be annihilated. Notably, this simplification assumes that all exciton pairs successfully split and does not consider recombination due to interfacial effects or internal defects. Thus, only two end points are possible: participation in the desired reaction or annihilation at an edge site.

We propose that whichever of these two endpoints is dominant depends on the time it will take for the hole to reach the relevant interface in either case. We will consider these times in turn.

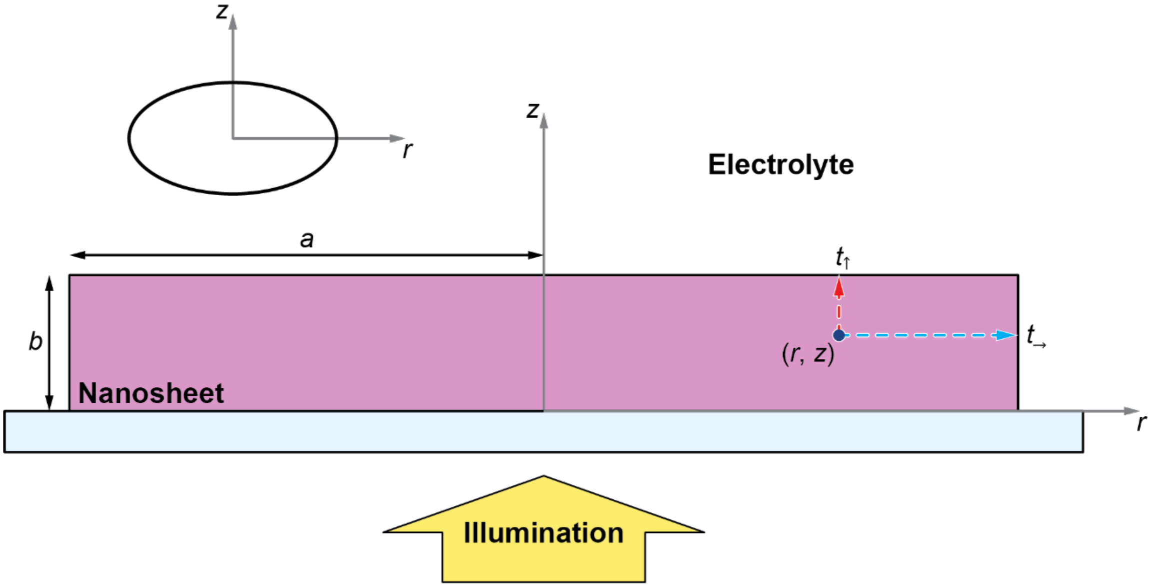

Figure S22. Schematic illustrating the system's geometry.

The time taken for the hole to diffuse roughly along the path of the dashed blue arrow to the edge of the nanosheet is given by: $t_{\rightarrow} = (a - r)^2/D_{\rightarrow}$, where $D_{\rightarrow}$ is the in-plane hole diffusion coefficient.

The time taken for the hole to drift to the nanosheet surface/electrolyte interface, roughly along the path of the dashed red arrow, is given by: $t_{\uparrow} = (b - z)/v_{\uparrow}$, where $v_{\uparrow}$ is the out-of-plane drift velocity. We assume the voltage drop across the nanosheet is V. Then, $v_{\uparrow} = \mu_{\uparrow}E = \mu_{\uparrow}V/b$, where $\mu_{\uparrow}$ is the out-of-plane hole mobility.

For any value of r, there will be a vertical position, z_c, where $t_{\rightarrow} = t_{\uparrow}$. At this point, either endpoint will be equally likely. However, for any point above this critical value of z (i.e., for $z > z_c$), it would be more likely that the hole will drift to the nanosheet surface/electrolyte interface. This means that the condition $t_{\rightarrow} = t_{\uparrow}$ defines the lower bound of the active region, where any photogenerated charges will reach the surface.

Setting these times to be equal ($t_{\rightarrow} = t_{\uparrow}$) yields: $(a - r)^2/D_{\rightarrow} = (b - z_c)b/\mu_{\uparrow}V$. Rearranging gives:

$$z_c = b - (a - r)^2\mu_{\uparrow}V/bD_{\rightarrow} \qquad \text{(S1)}$$

This equation determines the bottom edge of the active zone. In 3D space, it defines a surface. The active zone spans the volume above this surface, up to the nanosheet surface/electrolyte interface.

We can illustrate this by rearranging this equation slightly:

$$z_c/b = 1 - (1 - r/a)^2 a^2\mu_{\uparrow}V/b^2D_{\rightarrow}$$

and then plotting as a graph in the specific case where $a^2\mu_{\uparrow}V/b^2D_{\rightarrow} = 1$. This clearly shows the shape of the active zone as depicted in **Figure S23**.

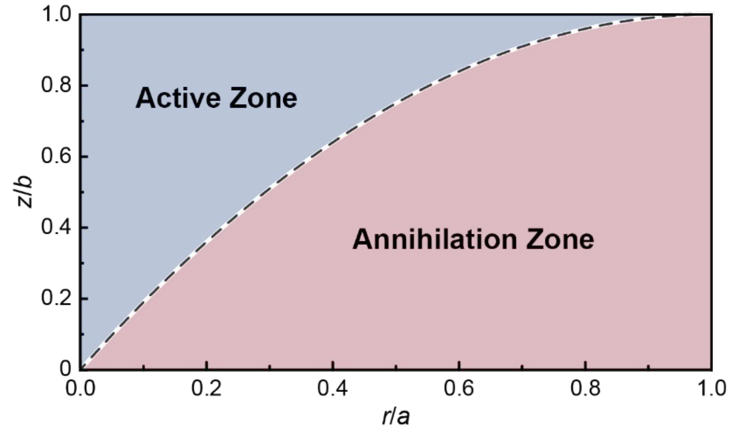

Figure S23. Plot showing the prediction of our model of the shape of the active zone (active zone lower boundary shown by the solid line), assuming $a^2\mu_{\uparrow}V/b^2D_{\rightarrow} = 1$.

3.2 Using the active zone boundary to calculate the photocurrent

Consider an infinitesimally small annular-shaped volume within the active zone. Every point within this volume is at a distance *z* above the electrode and a distance *r* from the center of the nanosheet (**Figure S24**).

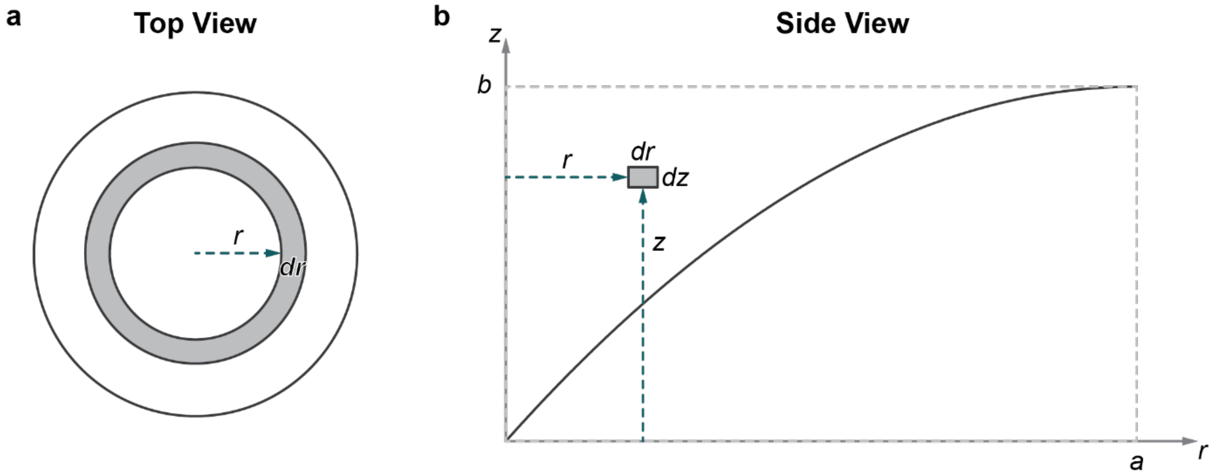

Figure S24. Schematic (a) top view, and (b) side view showing the annular integration volume $2\pi r dr dz$.

Assume the incident light intensity coming through the electrode is I_0, then the light intensity (W m^{-2}) at a vertical distance, *z*, inside the nanosheet is: $I = I_0 e^{-\alpha z}$.

The absorbed intensity within the thickness *dz* is:

$$\Delta I = I(z) - I(z+dz) = I_0(e^{-\alpha z} - e^{-\alpha(z+dz)}) = I_0 e^{-\alpha z}(1 - e^{-\alpha dz}) = I\alpha dz$$

The number of photons absorbed per unit area per second in *dz* is $dN_A = I\alpha dz/h\nu$. This means the number of photons absorbed per unit volume is $dN_V = I\alpha/h\nu$.

Thus, within our infinitesimal annular volume, the number of photons absorbed per second is dN_V multiplied by the annular volume ($2\pi r dr dz$):

$$dN = \frac{\alpha I(z)}{h\nu} \times 2\pi r dr dz \qquad \text{(S2)}$$

Here, we assume that all absorbed photons generate excitons and that all excitons generated within the active zone split to give an electron and a hole. We further assume that either the electron or the hole (depending on polarity) reaches the nanosheet surface electrode interface and participates in an electrochemical reaction. Under these assumptions, *dN* represents the number of charges generated per second in the annular volume $2\pi r dr dz$, which participate in the electrochemical reaction.

In order to calculate the overall charge generation rate associated with the entire active zone, one needs to integrate equation 2 over the whole volume of the active zone:

$$N = \frac{2\pi\alpha I_0}{h\nu}\int_0^a r dr \int_{z_c}^b e^{-\alpha z}\, dz$$

We note that the integral over the z-direction defines the fact that we are considering only excitons generated in the active zone. This means the limits of integration are the lower and upper spatial boundaries of the active zone, *i.e.,* z_c for the lower boundary (defining the interface between active and annihilation zones) and b for the upper boundary (defining the nanosheet surface-electrolyte interface). This approach assumes that the active/annihilation zone boundary is always within the nanosheet, *i.e.*, $z_c \geq 0$. This will be true so long as $b/a \geq \sqrt{\mu_{\uparrow}V/D_{\rightarrow}}$ (from Equation S1).

Performing the z-integration first:

$$N = \frac{2\pi\alpha I_0}{h\nu}\int_0^a rdr\left[\frac{-e^{-\alpha z}}{\alpha}\right]_{z_c}^b = -\frac{2\pi I_0}{h\nu}\int_0^a rdr\,[e^{-\alpha z}]_{z_c}^b$$

At this point, we realise the nanosheets are always very thin, which means we can make the approximation that $\alpha z \ll 1$ and so: $e^{-\alpha z} = 1 - \alpha z$. Then, applying this approximation:

$$N = -\frac{2\pi I_0}{h\nu}\int_0^a rdr\,[1 - \alpha b - 1 + \alpha z_c] = \frac{2\pi\alpha I_0}{h\nu}\int_0^a rdr\,[b - z_c]$$

Then, we can use equation 1 to replace z_c:

$$N = \frac{2\pi\alpha I_0}{h\nu}\int_0^a rdr\,[(a-r)^2\mu_{\uparrow}V/bD_{\rightarrow}] = \frac{2\pi\alpha I_0\mu_{\uparrow}V}{h\nu bD_{\rightarrow}}\int_0^a dr\,[a^2r - 2ar^2 + r^3]$$

Integrating over r gives:

$$N = \frac{2\pi\alpha I_0\mu_{\uparrow}V}{h\nu bD_{\rightarrow}}\left[\frac{a^2r^2}{2} - \frac{2ar^3}{3} + \frac{r^4}{4}\right]_0^a = \frac{2\pi\alpha I_0\mu_{\uparrow}Va^4}{h\nu bD_{\rightarrow}}\left[\frac{1}{2} - \frac{2}{3} + \frac{1}{4}\right] = \frac{\pi\alpha I_0\mu_{\uparrow}Va^4}{6h\nu bD_{\rightarrow}}$$

N is the number of electrons per second generated in the active zone. Assuming every one of these electrons reaches the nanosheet surface-electrolyte interface and participates in the electrochemical reaction, then the current density is given by $J = Ne/\pi a^2$:

$$J = \frac{e\alpha I_0\mu_{\uparrow}Va^2}{6h\nu bD_{\rightarrow}}$$

Rewriting slightly and noting that $\alpha I_0/h\nu = G_0$ is the maximum charge generation rate (i.e., that associated with incident, unattenuated radiation).

$$J_{ph} = \frac{ebG_0}{6}\left(\frac{a}{b}\right)^2\frac{\mu_{\uparrow}}{D_{\rightarrow}}V_{eff}$$

According to Einstein's relation, $D_{\rightarrow} = \mu_{\rightarrow}kT/e$. Where $\mu_{\rightarrow}$ is the in-plane hole mobility. Then

$$J_{ph} = \frac{e^2bG_0}{6kT}\left(\frac{a}{b}\right)^2\frac{\mu_{\uparrow}}{\mu_{\rightarrow}}V_{eff}$$ (**Equation 1**, Main Text)

We expect that in an electrochemical cell, the applied potential, ϕ, is related to the voltage drop across the nanosheet, V_{eff}, by: $\phi = V_{eff} + A$, where A is an unknown constant (units V). Then $dJ/dV_{eff} = dJ/d\phi$ and so:

$$\frac{dJ}{d\phi} = \frac{bG_0e^2}{6kT}\left(\frac{a}{b}\right)^2\frac{\mu_\uparrow}{\mu_\rightarrow} \qquad \text{(\textbf{Equation 2}, Main Text)}$$

We expect that the J-V curve will have a linear region (i.e., a constant slope) when the assumptions mentioned above all hold. However, as these assumptions break down (e.g., at high potential), the linearity will be lost, and the J-ϕ curve may saturate.

3.3 Writing the maximum carrier generation rate in terms of bandgap for broadband illumination (solar spectrum)

Above, we noted that $\alpha I_0/h\nu = G_0$ is the maximum carrier generation rate. However, in general, we will have a broadband source, so we must consider carrier generation over a range of frequencies. We need to incorporate the fact that both I_0 and α will often be frequency dependent.

For a broadband light source, we can write the total (integrated) intensity (W m^{-2}) as

$$I_0 = \int_0^\infty \frac{dI}{d\nu}d\nu \qquad \text{(S3)}$$

where $dI/d\nu$ is the spectral density of the source at the point of incidence on the sample (W m^{-2} Hz^{-1}). With this in mind, we can consider a broadband source characterized by an integrated intensity, I_0, incident on a very thin layer of material with an absorption coefficient that depends on frequency: $\alpha(\nu)$. If the layer is infinitesimally thin, then this represents the generation rate associated with the incident (non-attenuated) beam, i.e., the maximum rate. Then the rate of absorption of photons (s^{-1} m^{-3}) with frequency between ν and $\nu+d\nu$ is dG:

$$dG_0 = \frac{dI}{d\nu}\frac{\alpha(\nu)}{h\nu}d\nu$$

As mentioned above, this is equal to the maximum charge rate for photons in this range. Then the total (maximum) charge generation rate is:

$$G_0 = \int_0^\infty \frac{dI}{d\nu}\frac{\alpha(\nu)}{h\nu}d\nu$$

We can solve this equation under certain circumstances, considering a number of approximations. The solar spectrum incident on the sample can be modelled as proportional to the blackbody spectrum of the sun:

$$\frac{dI}{d\nu} = \frac{B\nu^3}{e^{h\nu/kT_\odot} - 1}$$

Where B is a constant and $T_{\odot}$ is the blackbody temperature of the sun. Because the integral of this function over all wavelengths is I_0, we can use equation 3 to calculate that $I_0 = (B/15)(\pi k T_{\odot}/h)^4$, allowing us to later relate B to the total integrated intensity (I_0, W m^{-2}) of the source incident on the sample.

The maximum charge generation rate is:

$$G_0 = \int_0^{\infty} \frac{B\nu^3}{e^{h\nu/kT_{\odot}} - 1} \frac{\alpha(\nu)}{h\nu} d\nu$$

Clearly, this can only be solved numerically once $\alpha(\nu)$ is known for the material under study. However, we can approximately solve this integral analytically by modelling $\alpha(\nu)$ as a step function, non-zero and constant (value α) only above the bandgap frequency: $\nu_g = E_g/h$. In practice, α is the average absorption coefficient over the energy range between the semiconductor band edge and the photon energy where the solar spectral irradiance becomes very small (3.5–4 eV). In addition, we use the Wein approximation for a blackbody spectrum. Combining these approximations:

$$G_0 = \frac{B\alpha}{h} \int_{E_g/h}^{\infty} \frac{\nu^2}{e^{h\nu/kT_{\odot}}} d\nu$$

This can be integrated by parts to yield

$$G_0 = \frac{B\alpha}{h} \left[\frac{{E_g}^2 kT_{\odot}}{h^3} + \frac{2E_g (kT_{\odot})^2}{h^3} + \frac{2(kT_{\odot})^3}{h^3} \right] e^{-E_g/kT_{\odot}}$$

Rearranging gives:

$$G_0 = \frac{B\alpha kT_{\odot}}{h^4} \left[{E_g}^2 + 2E_g kT_{\odot} + 2(kT_{\odot})^2 \right] e^{-E_g/kT_{\odot}}$$

Note that this function only changes by roughly ×3 when E_g is changed from 1.7 to 2.5 eV.

3.4 Final expression for current density in terms of bandgap

Then, combining all of the parts worked out above, we can obtain a general equation for the current density in a nanosheet illuminated with a broadband solar-like spectrum with an integrated intensity of I_0:

$$\frac{dJ_{ph}}{d\phi} = 15 I_0 (h/\pi k T_{\odot})^4 \frac{be^2}{6kT} \frac{\alpha k T_{\odot}}{h^4} \left[{E_g}^2 + 2E_g kT_{\odot} + 2(kT_{\odot})^2 \right] e^{-E_g/kT_{\odot}} \left(\frac{a}{b} \right)^2 \frac{\mu_{\uparrow}}{\mu_{\rightarrow}}$$

Simplifying:

$$\frac{dJ_{ph}}{d\phi} = \frac{5}{2} I_0 \frac{\alpha b e^2}{(\pi k T_{\odot})^3 \pi k T} \left(\frac{a}{b} \right)^2 \frac{\mu_{\uparrow}}{\mu_{\rightarrow}} \left[{E_g}^2 + 2E_g kT_{\odot} + 2(kT_{\odot})^2 \right] e^{-E_g/kT_{\odot}} \qquad \text{(S4)}$$

Parameters:

I_0 = Integrated intensity (W m^{-2}) of incident broadband (sunlike) beam

α = Average absorption coefficient of the semiconducting flake

b = Nanosheet thickness

a = Nanosheet radius

T = Sample temperature

$T_{\odot}$ = Blackbody temperature of the sun (5800 K)

$\mu_{\uparrow}$ = Out-of-plane mobility of charge carriers

$\mu_{\rightarrow}$ = In-plane mobility of charge carriers

E_g = Bandgap

ϕ = Electrode potential

3.5 Limitations of the model

While the model is in good agreement with experimental trends, it should be noted that this model simplifies the potential end points for both the excitons and minority charge carriers. Indeed, it assumes that all excitons will successfully split into free carriers and that the free carriers will either carry out the desired chemical reaction or be annihilated at an edge site. In reality, several outcomes are possible: (*i*) the exciton successfully splits into free carriers (e^- and h^+), (*ii*) the exciton remains bound (e^--h^+ pair), and (*iii*) the exciton binds to a free charge (trion, *e.g.*, e^--e^--h^+). Next, several outcomes are possible for the free charge carriers: (*i*) both charges are mobile and diffuse to the desired interface (reaction takes place), (*ii*) one or both charges are trapped (reaction may still take place), (*iii*) one or both charges recombine with another free charge (reaction may still take place), (*iv*) one or both charges recombine with another trapped charge (reaction may still take place), or (*v*) both charges are trapped or recombine (no reaction takes place). While this model considers that recombination is dominated by defects at edge sites, additional recombination and trap sites include internal defects and interfacial traps at the material/electrode or material/electrolyte interface.

Our previous works on commonly studied solution-processed TMDs such as MoS_2, WS_2, and WSe_2 have examined defect-mediated recombination of photogenerated charges. A combination of transient absorption spectroscopy (TA) and photoelectrochemical (PEC) measurements suggests that free charge carriers are the long-lived species and that defects at edge sites dominate recombination in these 2D material systems.[2-5] These works were done for one material system (*i.e.*, MoS_2 or WSe_2), wherein the effects of nanosheet dimensions could

be elucidated, showing that nanosheets with a higher ratio of edge to basal plane were more adversely affected. Notably, the work presented here explores materials with additional differences, including bandgap, in-plane, and out-of-plane charge mobility.

While fewer studies for the Sn-based dichalcogenides are available, preliminary work using transient Tetrahertz spectroscopy on chemical vapor-deposited SnS_2 nanosheets also identifies defects at edges as the primary site for recombination of photogenerated charges.[6] Thus, the assumption of this model that the dominant recombination mechanism is at defects at edge sites is reasonable. Future work using transient absorption and/or Terahertz spectroscopy will be necessary to build a more holistic picture of the range of materials presented in this work.

References

[1] Yu, J. *et al*. Ternary $SnS_{2-x}Se_x$ Alloys Nanosheets and Nanosheet Assemblies with Tunable Chemical Compositions and Band Gaps for Photodetector Applications. *Sci. Rep.* **5**, 17109 (2015).

[2] Yu, X. & Sivula, K. Photogenerated Charge Harvesting and Recombination in Photocathodes of Solvent-Exfoliated WSe_2. *Chem. Mater.* **29**, 6863–6875 (2017).

[3] Yu, X., Guijarro, N., Johnson, M. & Sivula, K. Defect Mitigation of Solution-Processed 2D WSe_2 Nanoflakes for Solar-to-Hydrogen Conversion. *Nano Lett.* **18**, 215–222 (2018).

[4] Morabito, F. *et al.* Long lived photogenerated charge carriers in few-layer transition metal dichalcogenides obtained from liquid phase exfoliation. *Nanoscale Adv.* **6**, 1074–1083 (2024).

[5] Tsokkou, D., Yu, X., Sivula, K. & Banerji, N. The Role of Excitons and Free Charges in the Excited-State Dynamics of Solution-Processed Few-Layer MoS_2 Nanoflakes. *J. Phys. Chem. C* **120**, 23286–23292 (2016).

[6] Zhang, W. *et al.* Ultrafast Photocarrier Dynamics in Vertically Aligned SnS_2 Nanoflakes Probing with Transient Terahertz Spectroscopy. *Nanomaterials* **13**, 5 (2023).
